# Integrating neglected tropical diseases into primary healthcare services in Ghana: Results from an initial pilot

**DOI:** 10.1371/journal.pntd.0014792

**Published:** 2026-10-07

**Authors:** Awurabena Quayeba Dadzie, Solomon Akwasi Ananya, Jean Jacques Ngamaleu Tougoue, Ruth Dede Tuwor, Michael Nkrumah-Appau, Joseph Kwadwo Larbi Opare, Ernest Mensah, Kwame Amankwah Bimpeh, Leslie Tetteh Gberbie, Abigail Agbanyo, Irene Dzathor, Bernadette Agbavor, Richard Odame Phillips, Yaw Ampem Amoako

**Affiliations:** 1 World Vision Ghana, Accra, Ghana; 2 World Vision USA, Federal Way, Washington, United States of America; 3 Kumasi Centre for Collaborative Research in Tropical Medicine, Kwame Nkrumah University of Science and Technology, Kumasi, Ghana; 4 Neglected Tropical Diseases Programme, Ghana Health Service, Accra, Ghana; 5 Centre for Health Information Management (CHIM), Policy Planning Monitoring and Evaluation Division, Ghana Health Service, Accra, Ghana; 6 FHI 360 Country Office, Accra, Ghana; 7 School of Medical Sciences, Kwame Nkrumah University of Science and Technology, Kumasi, Ghana; University of Sussex, UNITED KINGDOM OF GREAT BRITAIN AND NORTHERN IRELAND

## Abstract

**Background:**

Neglected Tropical Diseases (NTDs) affect billions globally, including a significant burden in Ghana where at least two NTDs are endemic in every district. Integrating NTD services into primary health care (PHC) is critical for achieving Universal Health Coverage (UHC) and meeting the 2021–2030 NTD roadmap goals. This study piloted the integration of Ghana Health Service NTD indicators into the Community-based Health Planning and Services (CHPS) home visit register in Ghana to assess the feasibility of this approach for national scale-up.

**Methods:**

A pilot implementation study was conducted in three districts representing different ecological zones in Ghana: Kassena Nankana West (northern), Nkwanta South (middle), and Ga South (southern). Following stakeholder workshops, the existing Community Health Officers’ (CHOs) home visit register was revised to include seven key NTD clinical indicators (blood in urine, skin lesions, visible deformities, eye complications, lymphoedema or swelling, suspected hydrocele and presence of a deep skin wound). A total of 64 Community Health Officers (CHOs) were trained to use the revised register during routine household visits over a three-month period (September to December 2023). Data on NTD cases identification, referrals, and referral compliance were collected and analyzed descriptively.

**Results:**

A total of 1,932 home visits were conducted across 34 health facilities resulting in 1,117 suspected cases (observations of NTD-related indicators) identified, 615 referrals made with 67% compliance and district level compliance ranging from 6.5% to 92.1%. CHOs successfully identified various NTD-related conditions, with distinct geographical patterns observed. Blood in urine was most frequently reported in Ga South, while lymphoedema and eye-related complications were more commonly reported in Kassena Nankana West. The varying patient compliance with the referrals suggests important contextual differences in linkage to care. Over 85% of trained health workers were actively implementing their new skills.

**Conclusion:**

Integrating NTD surveillance into the CHPS platform is feasible and yields valuable epidemiological data. However, the success of integration hinges not only on case detection but also on strengthening the linkage to care. The significant disparity in referral compliance highlights a critical health system bottleneck that must be addressed to ensure integrated services translate into improved health outcomes.

## Author summary

Neglected tropical diseases (NTDs) affect some of the world’s poorest and most vulnerable populations and remain a major public health challenge in Ghana, where multiple NTDs occur in every district. Integrating NTD services into routine primary health care is essential to improve early detection, referral, and access to care, and to support Ghana’s progress toward Universal Health Coverage. In this study, we explored whether NTD surveillance could be practically incorporated into the Community-based Health Planning and Services (CHPS) system, which delivers healthcare directly to households.

We piloted the inclusion of simple NTD indicators into the CHPS home visit register and trained Community Health Officers to use this tool during routine household visits in three districts across different ecological zones of Ghana. Using the revised register, health workers were able to identify large numbers of suspected NTD cases and generate valuable information on the geographical distribution of NTD-related conditions. Most trained health workers successfully applied their new skills in routine practice.

However, while case identification was feasible, many individuals who were referred to health facilities did not complete their referrals, with large differences observed between districts. Our findings show that integrating NTD surveillance into primary health care is achievable but improving referral pathways and addressing barriers to care are critical to ensure that early identification leads to better health outcomes for people affected by NTDs.

## Introduction

Neglected Tropical Diseases (NTDs) constitute a group of 21 diseases predominantly found in tropical regions characterized by insufficient access to clean water, sanitation, and hygiene infrastructure [1]. Currently, over 1.7 billion individuals globally are afflicted by NTDs, experiencing repercussions such as diminished productivity, disability, social stigmatization, and decreased socioeconomic and educational outcomes [2,3]. Although NTDs present considerable public health, social, and economic challenges, they are largely preventable and amenable to control and eventual elimination through coordinated public health interventions [4–7].

In Ghana, 14 endemic neglected tropical diseases (NTDs) have been identified, with at least two NTDs present in every district. The Preventive Chemotherapy (PC) NTDs include Onchocerciasis, Schistosomiasis, Soil-transmitted helminthiasis, Trachoma, and Lymphatic filariasis and Case Management (CM) NTDs include Dracunculiasis, Rabies, Human African trypanosomiasis, Yaws, Cutaneous leishmaniasis, Snakebite envenomation, Leprosy, Buruli ulcer, and Scabies. Schistosomiasis and soil-transmitted helminths (STH) are found in all districts [8]; leprosy in several districts; onchocerciasis in 53%; and lymphatic filariasis in 44% [9]. NTDs are highly prevalent in rural areas because of inadequate WASH systems [10–13]. These communities often rely on poorly equipped primary health care (PHC) systems [14]. Although trachoma has been eliminated as a public health problem in Ghana, eye-related indicators were retained in the surveillance package to support post-elimination surveillance and facilitate early detection of potential recrudescence.

In line with the NTD master plan and sustainability agenda, mainstreaming NTD services into existing health facility platforms will contribute to their control and elimination [9]. This will enable individuals with NTDs to have better access to all aspects of treatment, care, and support, leading to improved health outcomes. Such actions against NTDs contribute to achieving the Sustainable Development Goals (SDGs) set for 2030 [15]. The 2021–2030 roadmap for NTDs highlights the need for concerted multi-sectoral efforts toward the attainment of Universal Health Coverage (UHC) [1]. UHC is achieved when individuals and communities obtain essential health services without suffering financial hardships. The Sustainable Development Goal (SDG) target 3.8 envisions that countries *“achieve universal health coverage, including financial risk protection, access to quality essential health-care services, and access to safe, effective, quality, and affordable essential medicines and vaccines for all”* [16]. Mainstreaming NTDs into PHC is critical for achieving UHC and ensuring the provision of equitable health access and adequate data for decision-making. Furthermore, it will also contribute to reducing stigma, easing financial burdens, enhancing food security, and improving economic development.

Significant data gaps for NTDs arise from limitations in reporting systems, equipment availability, and case reporting. Large data gaps exist for NTDs due to deficiencies in reporting systems [17,18]. WHO recommends reporting on all relevant NTDs and disaggregated data for NTDs to improve country ownership on NTD interventions for informed health policies towards Universal Health Coverage (UHC) and improved health outcomes [1].

Community Health Officers are formally trained frontline health professionals employed by the Ghana Health Service and are responsible for delivering community-based primary health care services through the Community-based Health Planning and Services (CHPS) programme. In Ghana, Community Health Officers (CHOs) utilize the home visit register during household visits to collect community-level health data. Data routinely collected in the home visit register include information on maternal and child health services (antenatal, postnatal care immunization and family planning services), nutrition and health promotion (identification of severe acute malnutrition, growth monitoring and promotion), wellness (Blood pressure, body mass index) [19,20]. The register was revised to include NTD indicators to facilitate mainstreaming of NTD services into the primary health care system of Ghana. The electronic and paper-based versions of the revised home visit register were piloted in three districts: Kassena Nankana West, Nkwanta South and Ga South.

The primary goal of this pilot study was to assess the feasibility of integrating selected NTD-related syndromic indicators into the routine primary health care using Community-Based Health Planning and Services (CHPS) home visit register in chosen districts by collaborating with stakeholders at national, sub-national, and district levels. We evaluated identification, referral, and referral-completion processes for suspected cases.

## Methods

### Ethics statement

This study presents the outcome of an implementation activity under the auspices of the Ghana Health Service. Approval was obtained from the Committee on Human Research and Publication Ethics of the School of Medical Sciences of the Kwame Nkrumah University of Science and Technology (approval number: CHRPE/AP/335/19). Further, permission was sought from local authorities and heads of health facilities. All participants provided written informed consent. For participants younger than 18 years, written informed consent was obtained from a parent or legal guardian, with additional assent obtained from children 12 – 17 years. To facilitate referral and follow-up, limited personal identifiers, including participant name, age, sex, community of residence, contact information, and referral identification number, were collected and recorded on referral forms. For individuals less than 18 years old, the name and contact details of a parent or guardian were also recorded where necessary. These identifiable data were used solely for the purpose of clinical referral and care coordination and were accessible only to authorized study and health service personnel. All data were de-identified prior to analysis and reporting.

### Study setting

The implementation study was conducted in 3 selected districts in Ghana namely, Kassena Nankana West (KNW) in the northern zone, Nkwanta South (NS) in the middle zone and Ga South (GS) district in the southern zone. Within each district, two subdistricts were chosen for the study. The districts and subdistricts, located in the northern, middle and southern zones of Ghana were selected in consultation with the Neglected Tropical Diseases (NTD) Control Programme of the Ghana Health Service based on their level of NTD endemicity and to ensure balanced geographical representation across the country. The choice of districts was intended to provide illustrative implementation settings rather than a nationally representative sample.

Most members of the population in these districts are engaged in agriculture, forestry and fishing jobs [21,22]. The KNW has a population of 90,735 according to the 2021 National Population and Housing Census (NPHC) [23]. The district is within the Upper East region, and the population has an almost equal distribution of males and females [21].

The Nkwanta South district is located in the Oti region of Ghana and has a population of 135,936 which is evenly distributed between the sexes [23,24]. The Municipality has a population density of 35 people per square kilometre [25]. The Nkwanta South Municipality consists of 6 sub-districts.

Ga South Municipality is found in the Greater Accra region of Ghana. The Municipality occupies a total land area of 341.838 square kilometres and is made up of 20 sub-districts [26]. The total population of the Municipality is approximately 485,643 and consists of 248,085 (51.1%) females [27,28].

The study districts are mainly rural and most community members have limited access to healthcare [29].

### Definitions of terms

Indicators were developed by experts from the Ghana NTD control programme, clinicians, Intra-country Coordinating Committee for NTDs and other partners during Step I of the implementation phase. The following operational definitions were used as proxy indicators for NTDs:

- *Skin lesions: hypopigmented or red patches, nodules and skin infiltrations*
- *Hydrocele: any scrotal swelling (in males)*
- *Visible deformities: visible deformities such as clawing, shortening of digits, limitation of joint movement*
- *Eye complications: reddening, tearing and weakness or loss of eye structures*
- *Deep skin wounds: Any wounds/ ulcers with undermined edges and slough*
- *Lymphoedema: swollen limbs in persons*
- *Blood in urine: self-reported passage of blood in urine*

### Implementation

The details of the steps taken in the implementation are shown in Fig 1. The steps included technical workshops, sub-national sessions, training of field teams on piloting tools, roll out of pilot and monitoring and evaluation.

**Fig 1:**
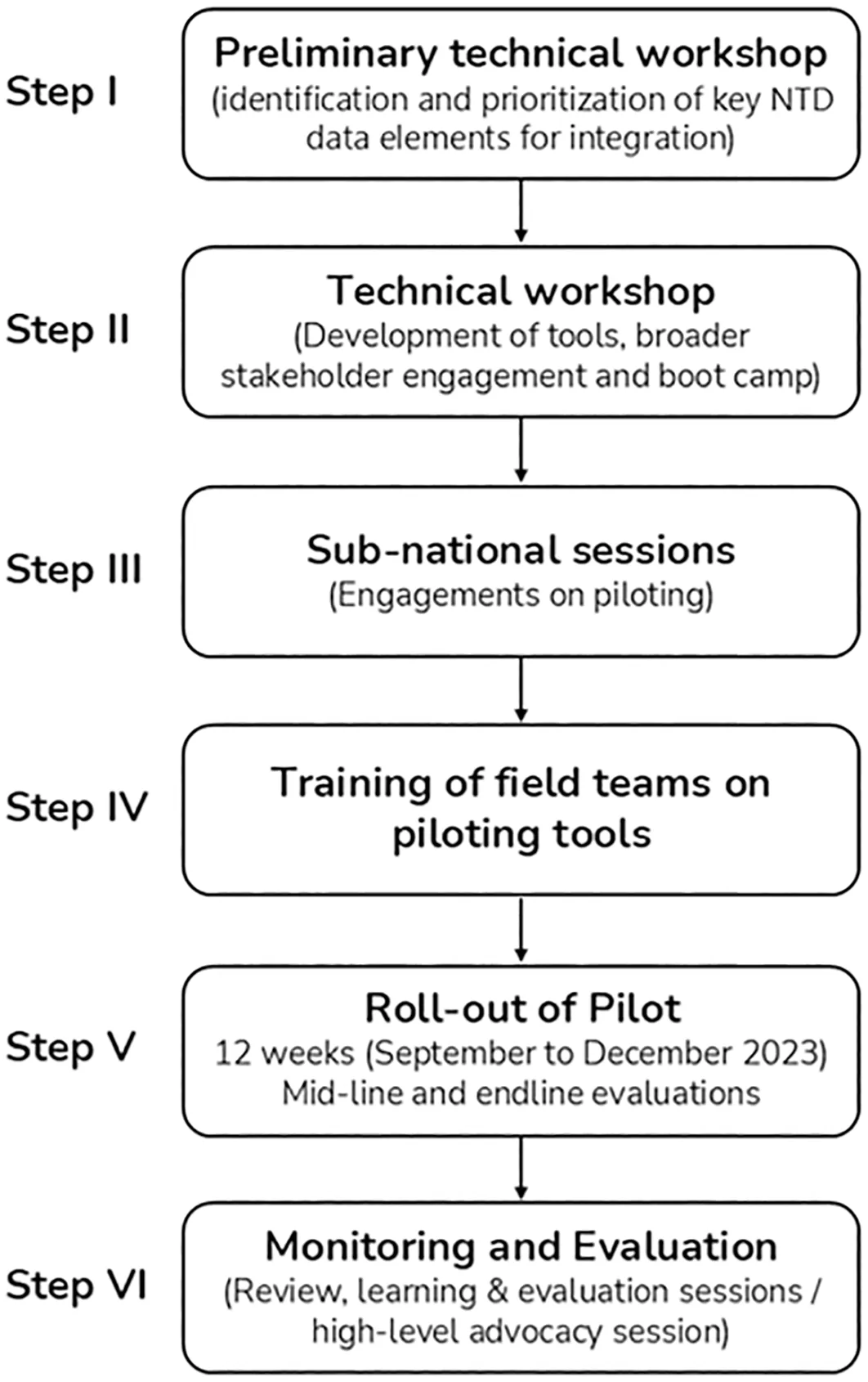
Steps in study implementation.

#### Technical workshop.

This workshop was held over 5 days and aimed to identify and prioritize key NTD data elements for integration/ mainstreaming into the CHO home visit register. Participants were from the Neglected Tropical Diseases Programme and Centre for Health Information Management Unit of the Ghana Health Service, Intra-Country Coordination Committee for NTDs (ICCC) and other stakeholders such as World Vision Ghana (WVG). Another key outcome of these engagements was the identification of the home visit register used by CHOs as the entry point to integrate NTDs into routine Primary health care (PHC) services in Ghana. Following a tool development workshop and stakeholder consultation, the existing home visit register was modified to include selected NTD indicators. Subsequently, a bootcamp was held with technical support from the Centre for Health Information Management (CHIM), a department under the Policy Planning, Monitoring and Evaluation Division of the Ghana Health Service to programme and test the identified data elements into the data collection tool. This NTD-integrated home visit register was agreed to be piloted across the three districts.

#### Sub-national sessions/ Training of field teams on piloting tools.

The NTD Control programme was supported by partners (WVG) to conduct a two-day healthcare worker training in each of the implementation districts. The training was held to equip health care workers with the technical know-how to conduct effective piloting of the NTD-integrated home visit register, in aspects of effective home visiting and reporting. Participants were trained on identifying the NTD indicators, as well as basic software skills to facilitate use of electronic data collection. A total of 64 Community Health Officers (Kassena Nankana = 21; Ga South = 25; Nkwanta South = 18) were trained. In addition, 23 Disease Control officers, Public Health nurses and Health information officers from the districts were trained to monitor and provide supervision oversight during implementation.

#### Roll out of pilot.

CHOs in communities within the study districts implemented the actual roll out of the revised home visit register over a 3-month period (September to December 2023). CHOs were trained using standardized case definitions, visual aids, and practical exercises to recognize the seven selected NTD-related indicators. Household visits were conducted as part of routine outreach activities by CHOs within their assigned CHPS catchment areas. No predefined population sampling frame or population-level denominator was established because the pilot was designed to evaluate operational feasibility rather than estimate disease prevalence. During their household visits, CHOs screened available household members using the revised register and recorded the presence or absence of the predefined NTD-related indicators as captured in the new register. During each household visit, all household members who were present at the time of the visit and consented to participate were screened for the seven NTD indicators. Individuals who were absent during the visit were not assessed unless encountered during subsequent routine visits. As this was a feasibility and implementation pilot, no formal sample size calculation was conducted. The number of observations reflects routine implementation activities undertaken during the study period. Referral compliance was assessed through routine follow-up visits conducted by CHOs and district monitoring teams using referral records and documented evidence of attendance at the designated health facilities within the implementation period. Referral compliance was defined as the proportion of referred individuals who had documented evidence of attending the designated health facility within the study follow-up period. The denominator for referral compliance comprised all individuals referred from community screening, while the numerator included those with verified facility attendance records. Individuals without documented attendance were classified as non-compliant for the purposes of this analysis.

#### Monitoring and evaluation.

In each district, monitoring was carried out by a team drawn from the NTD control programme, CHIM, and the staff of the District Health Directorates (including Disease control officers, public health nurses, health information officers). During the monitoring visits, observations were made on how data was collected in the home visit register and CHOs were interviewed. Data was collected on training and capacity building of CHOs, effectiveness of training on service delivery and assessment of health facilities in NTD education and service delivery. In addition, the monitoring tool assessed the electronic and paper-based interface and its user-friendliness, as well as home visits and referrals. Data was also collected on NTD indicators which were identified during home visits. Furthermore, data quality in terms of availability, timeliness, consistency, and completeness was collected and recommendations and success stories were documented by the monitoring team.

#### Revised CHPS home visit register.

The revised CHPS home‑visit register was designed to capture observable clinical signs associated with endemic neglected tropical diseases, consistent with World Health Organization guidance on integrated, community‑based surveillance. The seven indicators – blood in urine, skin lesions, visible deformities, eye complications, lymphoedema or swelling, suspected hydrocele, and deep skin wounds – map to multiple priority NTDs targeted under WHO control and elimination frameworks (Fig 2). Specifically, blood in urine which may suggest urogenital schistosomiasis; lymphoedema, hydrocele, and visible deformities are characteristic of chronic lymphatic filariasis; skin lesions and eye complications may reflect manifestations of onchocerciasis and trachoma; while skin lesions and deep skin wounds are consistent with yaws, scabies, and leprosy‑related morbidity. Collectively, these indicators enable frontline health workers to identify suspected cases of several preventive‑chemotherapy and morbidity‑focused NTDs during routine household visits, supporting early detection and referral within the primary health care system.

**Fig 2:**
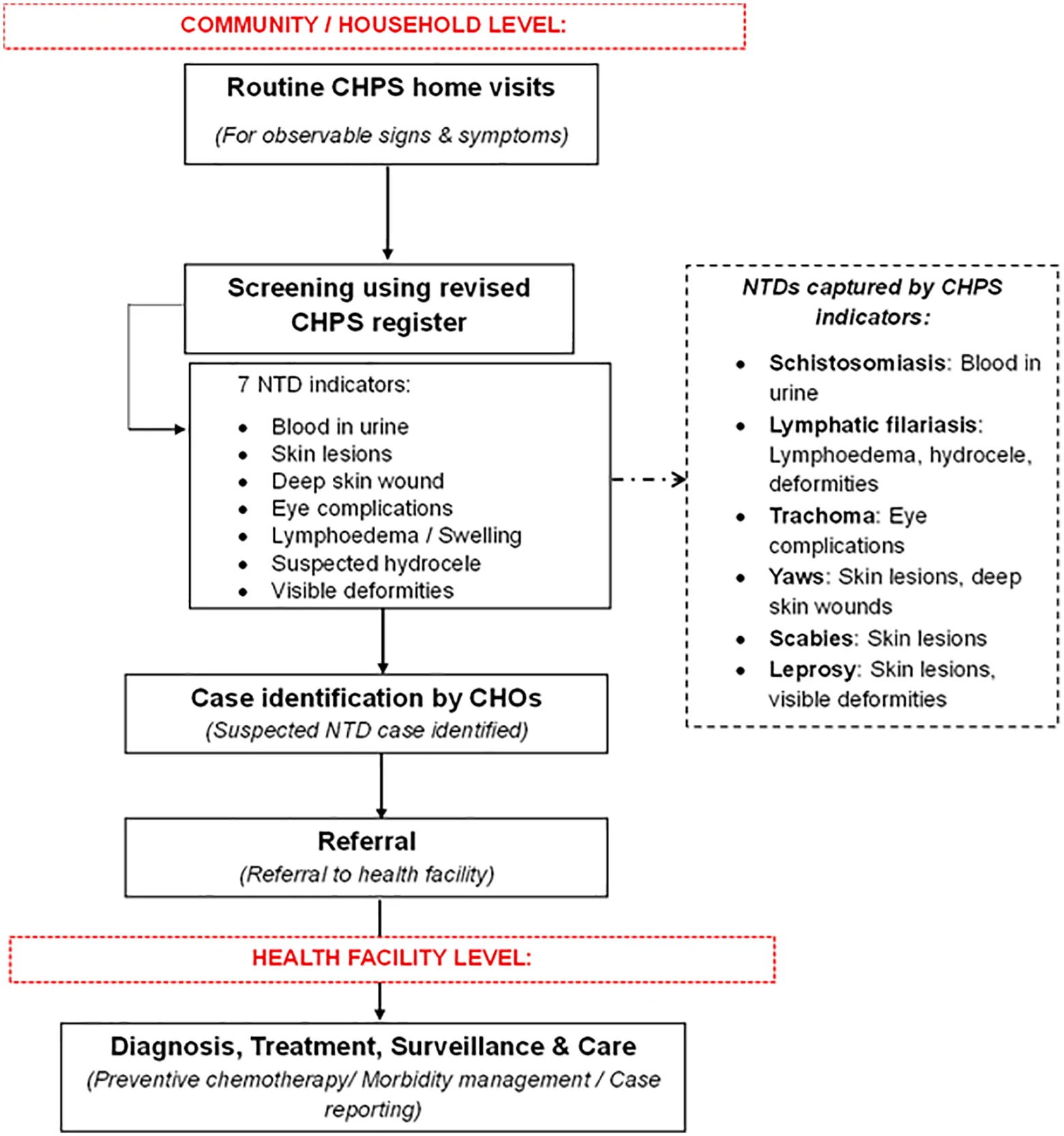
Conceptual framework linking Community‑based surveillance for Neglected Tropical Diseases to primary health care delivery through the CHPS system in Ghana. During routine household visits, Community Health Officers screen individuals for seven observable NTD indicators using a revised CHPS home‑visit register. These indicators correspond to the characteristic clinical manifestations of several endemic NTDs aligned with WHO control and elimination frameworks. Suspected cases trigger referral to health facilities for confirmation, treatment – including preventive chemotherapy where applicable – and reporting. The framework illustrates how symptom‑based community screening can strengthen early detection, surveillance, and linkage to care within an integrated primary health care system.

### Data management and statistical analysis

The data were abstracted into Microsoft Excel 2016 (Microsoft Corp., Redmond, WA, USA) and analyzed using Stata version 12 (Stata Corp LP, College Station, Texas, USA). We performed a descriptive analysis of demographic and clinical variables. The analysis was primarily descriptive and focused on assessing implementation feasibility, uptake, indicator detection, referral patterns, and referral completion. No formal hypothesis-testing comparisons between districts were planned. Figures were generated using GraphPad Prism version 6.0 (GraphPad Software, San Diego, California USA).

## Results

### Characteristics of the study sample

A total of 34 health facilities across three districts in Ghana participated in the pilot implementation of the NTD-integrated home visit register. These comprised 11 facilities (32.4%) in Kassena Nankana West (KNW) district, 9 facilities (26.4%) in Nkwanta South (NS) district, and 14 facilities (41.2%) in Ga South district (Table 1). The facilities included a mix of CHPS compounds and health centres, reflecting the typical primary health care landscape in Ghana.

**Table 1:** Health facilities within the study districts.

District	Number of facilities, n (%)	Health facilities
Kassena-Nankana West (KNW)	11 (32.4)	• Amutanga CHPS • Gunwoko CHPS • Basengo CHPS • Bugsongo CHPS • MOU HC • Wugingo CHPS • Nyangolingo CHPS • Kuliya CHPS • St. Francis Assisi Health Centre • St. Joseph Health Centre - Nakolo • Boania CHPS
Nkwanta South (NS)	9 (26.4)	• Salifu CHPS • Bontibor CHPS • Pusupu CHPS • Brewaniase Health Centre • Abrubruwa CHPS • Obanda CHPS • Kecheibi CHPS • Tutukpene CHPS • Ofosu CHPS
Ga South	14 (41.2)	• Akweiman CHPS • Osofo Lamptey CHPS • Kwame Anum CHPS • Balagonnor CHPS • Hobor CHPS • Kwaku Pamfo CHPS • Horni CHPS • Kofi Kwei CHPS • Langma CHPS • Jei Krodua CHPS • Oshiyie CHPS • Lai Gonno CHPS • Tuba CHPS • Obom CHPS

Across these facilities, 64 Community Health Officers (CHOs) were trained to use the revised register, with district-specific training numbers as follows: KNW (n = 21), GS (n = 25), and NS (n = 18).

### NTD-related clinical observations

During the three-month pilot period, CHOs conducted a total of 1,932 home visits and successfully recorded 1,117 observations for seven pre-defined NTD-related clinical indicators; individual participants could contribute more than one indicator observation. The frequency of these indicators showed marked geographical variation across the three districts. *Skin lesions* were the most common NTD-related clinical indicator reported across all three study sites. Ga South accounted for 59.3% (386/651) of all recorded skin lesion observations, followed by Nkwanta South with approximately 32.9% (Table 2).

**Table 2:** Distribution of recorded syndromic NTD indicators across study districts.

District	Clinical conditions *n (%)*						
	Skin lesions	Visible deformities	Eye complications	Lymphoedema	Suspected hydrocele	Deep skin wound	Blood in urine
Ga South	386 (59.3)	62 (81.6)	38 (31.7)	29 (17.4)	17 (40.5)	17 (41.5)	15 (75.0)
Nkwanta South	214 (32.9)	0 (0.0)	1 (0.8)	3 (1.8)	8 (19.0)	21 (51.2)	1 (5.0)
Kassena Nankana West	51 (7.8)	14 (18.4)	81 (67.5)	135 (80.8)	17 (40.5)	3 (7.3)	4 (20.0)
Total	651 (100.0)	76 (100.0)	120 (100.0)	167 (100.0)	42 (100.0)	41 (100.0)	20 (100.0)

*Percentages represent the proportion of all recorded observations for each indicator contributed by each district and not disease prevalence estimates within the districts.

Ga South accounted for 81.6% (62/76) of *visible deformities* followed by Kassena Nankana West (18.4%), with no cases documented in Nkwanta South (Table 2). The district located in Northern Ghana, Kassena Nankana West, accounted for 81 of 120 (67.5%) recorded eye-related complications and 135 of 167 (80.8%) recorded lymphoedema observations.

*Hydrocele* was equally (40.5%; 17/42) common in the Ga South and Kassena Nankana West districts. *Deep skin wounds* were most commonly reported from Nkwanta South (51.2%; 21/41) and Ga South (41.5%; 17/41) districts.

Notably, the coastal Ga South district accounted for 75% (15/20) of the total recorded observations of the presence of *blood in urine*, a key indicator which may suggest urogenital schistosomiasis.

### Referrals and linkage to care

A total of 1,932 home visits were conducted across the three districts, with Kassena Nankana West recording the highest number of visits (851), followed by Ga South (672) and Nkwanta South (409). During these visits, 1,117 indicators requiring attention were identified, comprising 564 in Ga South, 248 in Nkwanta South, and 305 in Kassena Nankana West (Table 2). Referral proportions were calculated as the number of referrals generated per household visit conducted in each district. Because multiple individuals may be screened during a single household visit, these proportions reflect referrals relative to household visits rather than the proportion of individuals referred. The proportion of referrals relative to household visits varied substantially across districts. Nkwanta South recorded the highest referral rate, with 248 of 409 visits (60.6%; 95% CI: 55.8–65.2%) leading to a referral. Kassena Nankana West followed with 305 of 851 visits (35.8%; 95% CI: 32.7–39.1%), while Ga South had the lowest referral rate, with 62 of 672 visits (9.2%; 95% CI: 7.2–11.6%). Among those referred, attendance at a health facility also differed markedly by district. Kassena Nankana West achieved the highest referral completion rate, with 281 of 305 referred cases (92.1%; 95% CI: 88.5–94.7%) attending a health facility. Nkwanta South recorded an attendance rate of 52.4% (130/248; 95% CI: 46.2–58.6%), whereas Ga South had the lowest attendance rate, with only 4 of 62 referred cases (6.5%; 95% CI: 2.0–15.7%) attending a health facility (Table 3). Overall, while Nkwanta South demonstrated the highest rate of referral generation, Kassena Nankana West showed the strongest linkage between referral and actual health facility attendance. In contrast, Ga South recorded both the lowest referral rate relative to household visits and the lowest proportion of referred individuals who subsequently attended a health facility.

**Table 3:** Distribution of home visits, referrals and linkage to care across districts.

Activity	District					
	`Ga South	*95% CI*	Nkwanta South	*95% CI*	Kassena Nankana West	*95% CI*
Total number of home visits (n)	672		409		851	
Total indicators identified (n)	564		248		305	
Proportion of home visits leading to referrals (n/N, %)	62/672 (9.2%)	*7.2–11.6*	248/409 (60.6%)	*55.8–65.2*	305/851 (35.8%)	*32.7–39.1*
Proportion of referrals resulting in actual attendance at health facility (n/N, %)	4/62 (6.5%)	*2.0–15.7*	130/248 (52.4%)	*46.2–58.6*	281/305 (92.1%)	*88.5–94.7*

### Training and Implementation by health workers

A total of 87 healthcare workers (including CHOs and supervisory staff) were trained across the three districts as part of the pilot preparations; however, the implementation assessment was conducted only among cadres (CHOs, public health nurses and disease control officers) directly responsible for carrying out community screening and referral activities during the period. The trained workers who were not directly involved in implementation activities and/or were not available for follow-up assessment during the evaluation period were not included in this analysis. Post-implementation monitoring assessed the extent to which these workers were applying their newly acquired knowledge and skills during routine duties. Overall training implementation among health workers was high across the districts with more than 85% of trained workers were actively implementing the NTD identification and reporting protocols. Facility-level performance was highest in Kassena Nankana West, where 100% (21/21) of trained community health officers (CHOs) were observed to be implementing the training. In Ga South, 21/25 (84.0%) of trained CHOs implemented the training while in Nkwanta South, implementation was reported among 83.3% (15/18) of the trainees (Fig 3).

**Fig 3:**
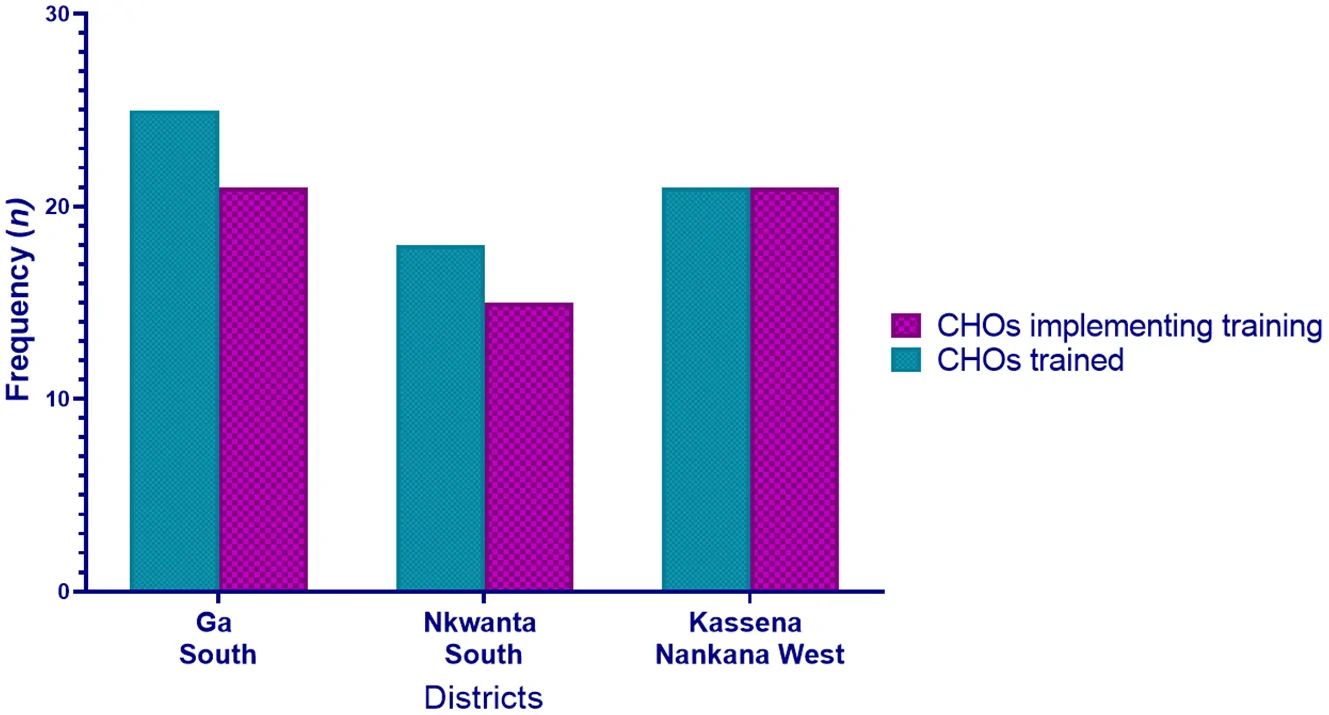
Proportion of community health officers implementing training received.

Review of both the electronic and paper registers used in parallel during the piloting showed that no major discrepancies affecting interpretation were identified during the monitoring activities.

## Discussion

This pilot study provides compelling evidence that integrating Neglected Tropical Disease (NTD) services into Ghana’s primary health care (PHC) system, specifically the Community-based Health Planning and Services (CHPS) platform, is not only feasible but also generates valuable, locally relevant data. By embedding NTD indicators into the routine home visit register, we have demonstrated a pragmatic pathway toward operationalizing the World Health Organization’s 2021–2030 NTD roadmap and advancing Universal Health Coverage (UHC). The data collected by Community Health Officers (CHOs) revealed distinct patterns - such as the high frequency of blood in urine in the coastal Ga South district and lymphoedema in the northern Kassena Nankana West district - demonstrating the ability of CHOs to act as effective surveillance sentinels to identify and report syndromic indicators. While the diagnostic accuracy of the CHOs was not formally assessed, this community-level intelligence is crucial for filling the significant data gaps highlighted in Ghana’s NTD master plan [9] and enables a shift from uniform interventions toward targeted, equitable resource allocation. The high rate of knowledge implementation among trained CHOs further underscores the potential sustainability of this integrated model, transforming them into more comprehensive primary health care practitioners.

The integration of NTD-related syndromic screening into the CHPS platform complements existing NTD control strategies in Ghana by strengthening community-based case detection and referral pathways for individuals requiring further assessment and care. The identification of clinical signs such as hydrocele, lymphoedema, and visible deformities creates a formal pathway for patients to access morbidity management and disability prevention services, as well as surgical interventions. This addresses a critical gap in the NTD care continuum. Substantial variation was observed in the proportions of skin lesions and visible deformities across districts. While Nkwanta South reported a higher proportion of skin lesions without visible deformities, Kassena Nankana West and Ga South recorded higher proportions of visible deformities. This pattern may reflect differences in the timing of case detection, health-seeking behaviour, community awareness, referral pathways, or local case-finding activities. In particular, the high proportion of visible deformities in some districts may be influenced by the small number of reported cases, resulting in unstable percentages. Since information on healthcare access, health facility capacity, and diagnostic delays was not assessed, the underlying causes of these district-level differences could not be determined and warrant further investigation.

The pilot revealed a major implementation bottleneck: the “referral-compliance gap.” While several home visits in some districts resulted in a referral, actual patient attendance at health facilities was low in two of the three districts. Referral compliance varied markedly across districts, ranging from 92.1% in Kassena Nankana West to 52% in Nkwanta South and less than 10% in Ga South. These differences may reflect variations in healthcare access, transportation barriers, socioeconomic factors, community awareness, stigma, health-seeking behaviour, and the effectiveness of referral follow-up systems. While Kassena Nankana West may have benefited from stronger community-facility linkages and patient follow-up, barriers to linkage-to-care may have contributed to the lower compliance observed in Nkwanta South and Ga South. As our study did not investigate determinants of referral completion, further research is needed to identify district-specific factors associated with referral compliance. Our findings suggest that passive referral systems are insufficient. It implies that simply detecting a suspected NTD case at the community level does not guarantee that the patient will navigate the barriers of distance [30], cost [3], stigma [31], or lack of awareness to reach a diagnostic or treatment center [32]. For integration to be truly effective, it must be coupled with active strategies to bridge this gap. Strengthening facility-level capacity, availability of clinical guidelines, training of healthcare workers, and access to essential diagnostic and treatment services would be critical next steps for achieving broader NTD integration and Universal Health Coverage.

The inclusion of districts from Ghana’s northern, middle, and southern zones enhances the diversity of contexts represented in the study, providing preliminary evidence on the intervention’s applicability across varied ecological and socio-cultural settings. The success in the rural Kassena Nankana West district, where CHOs achieved 100% implementation and high referral compliance, suggests the model is particularly well-suited to remote areas with established, trusted community health structures. Conversely, the challenges encountered in the more peri-urban Ga South district highlight that integration strategies may require contextual adaptation. The lower referral compliance in this setting may reflect different patient priorities, greater availability of alternative health providers, or urban-specific barriers like transportation costs within a large municipality. While the pilot focused on NTDs with visible signs, the findings suggest that task-sharing surveillance to CHOs could have broader applicability; however, further research is needed to determine its effectiveness and feasibility in other contexts. This same platform could be readily adapted to screen for other NTDs like Buruli ulcer, or to monitor for adverse events. Furthermore, this integrated model, if confirmed in larger studies in similar settings, can offer a valuable template for other countries in sub-Saharan Africa that are leveraging community health worker networks to achieve UHC and meet NTD elimination goals.

A key strength of this study is its pragmatic, implementation-focused design. By engaging national, regional, and district-level stakeholders from the outset through technical workshops, we fostered a sense of co-ownership, which is critical for future policy uptake and national scale-up. The monitoring approach assessed both quantitative outputs (signs, referrals) and incorporated qualitative feedback on tool usability, thereby providing a comprehensive understanding of the implementation process. However, several limitations must be acknowledged. First, the three-month pilot period may not capture seasonal disease variations, such as the peak transmission periods for schistosomiasis. Second, the data represent “suspected” cases based on clinical syndromic observation by CHOs, without confirmatory diagnostics, which could lead to misclassification. The use of ‘syndromic’ indicators that intentionally prioritised sensitivity over diagnostic specificity could possibly result in misclassification. No population-level denominator was available, preventing estimation of screening coverage. Furthermore, the descriptive nature of the study design and lack of population denominators, and small sample sizes for some indicators limit generalizability. Some observations were based on relatively small numbers and should therefore be interpreted cautiously. Additionally, because facility-based diagnostic confirmation data were not available, the positive predictive value of CHO-based identification could not be assessed. Also, while we documented the referral compliance gap, the study design did not include a qualitative component to explore the reasons behind patients’ decisions to seek or not seek further care. This limits our ability to design targeted interventions to address this bottleneck. Finally, the pilot’s short duration does not allow for an assessment of the long-term sustainability of data quality or CHO motivation.

The findings of this pilot have immediate and actionable implications for the Ghana Health Service. The demonstrable feasibility and high uptake by CHOs can support the consideration of a phased national roll-out of the revised home visit register. To address the critical referral-compliance gap, district health managers should implement targeted interventions. These could include establishing a patient navigation system, providing transport subsidies for referred patients with suspected NTDs, or conducting community sensitization dialogues to address stigma and build trust in the formal health system. Learning from the high-performing Kassena Nankana West district through “audit and feedback” visits could provide valuable peer-to-peer insights for lower-performing areas. Future research should employ implementation science frameworks to prospectively identify barriers and enablers of referral compliance from both the patient and health system perspectives. A cost-effectiveness analysis comparing this integrated model to the previous vertical approach would provide robust economic evidence for policymakers. Finally, future iterations should explore enhancing the electronic version of the register with digital decision-support tools to aid CHOs in differential diagnosis and provide standardized, algorithm-based management protocols, further strengthening the critical link between community detection and quality care.

## Conclusion

This pilot demonstrates that integrating Neglected Tropical Diseases (NTDs) into Ghana’s primary health care system through the CHPS platform is feasible and operationally practical. Incorporating simple NTD indicators into routine home visit registers enabled community health officers to detect suspected cases and generate useful, location-specific data across diverse settings. Our findings provide proof-of-concept operational evidence demonstrating the feasibility and acceptability of incorporating syndromic NTD indicators into routine CHPS activities, the strong engagement of Community Health Officers, and the importance of addressing gaps in referral completion. However, large disparities in referral rates and referral compliance underscore persistent health system and community-level barriers to effective linkage to care that could limit health impact. Addressing community- and system-level barriers to referral completion will be essential. Overall, integrating NTD services into PHC offers a promising pathway toward Universal Health Coverage and achievement of Ghana’s NTD control and elimination goals, provided that detection is matched by timely and effective care. Our findings support the need for larger studies to evaluate effectiveness, implementation requirements, and scalability in diverse settings.

## References

[1] WHO. Ending the neglect to attain the sustainable development goals: A rationale for continued investment in tackling neglected tropical diseases 2021–2030: World Health Organization; 2022.

[2] Gyapong M, Immurana M, Manyeh A, Odopey CT, Dean L, Krentel A. The social and economic impact of neglected tropical diseases in sub-Saharan Africa. PLoS Negl Trop Dis. Springer. 2024. p. 479–503.

[3] Hailemichael Y, Novignon J, Owusu L, Okyere D, Mtuy T, Alemu AY, et al. The role of economic factors in shaping and constituting the household burden of neglected tropical diseases of the skin: Qualitative findings from Ghana and Ethiopia. Soc Sci Med. 2024;356:117094. doi: 10.1016/j.socscimed.2024.117094 39032192 PMC11370647

[4] Casulli A. New global targets for NTDs in the WHO roadmap 2021–2030. PLoS. 2021. p. e0009373. 10.1371/journal.pntd.0009373 PMC8118239 33983940

[5] Conteh L, Engels T, Molyneux DH. Socioeconomic aspects of neglected tropical diseases. Lancet. 2010;375(9710):239–47. doi: 10.1016/S0140-6736(09)61422-7 20109925

[6] Molyneux DH. Neglected tropical diseases: Now more than just ’other diseases’—the post-2015 agenda. Int Health. 2014;6(3):172–80. doi: 10.1093/inthealth/ihu037 24969646

[7] Mitra AK, Mawson AR. Neglected tropical diseases: Epidemiology and global burden. Trop Med Infect Dis. 2017;2(3):36. doi: 10.3390/tropicalmed2030036 30270893 PMC6082091

[8] Boateng EM, Dvorak J, Ayi I, Chanova M. A literature review of schistosomiasis in Ghana: A reference for bridging the research and control gap. Trans R Soc Trop Med Hyg. 2023;117(6):407–17. doi: 10.1093/trstmh/trac134 36688317

[9] GHS. Ghana NTD master plan 2021-2025. Ghana Health Service; 2021 [cited 2026 Mar 12]

[10] Gillis S. Water, sanitation, and hygiene (WASH) services among households, healthcare facilities and schools in rural Ghana. Yale University. 2025.

[11] Johnston EA, Teague J, Graham JP. Challenges and opportunities associated with neglected tropical disease and water, sanitation and hygiene intersectoral integration programs. BMC Public Health. 2015;15(1):547. doi: 10.1186/s12889-015-1838-7 26062691 PMC4464235

[12] Campbell SJ, Biritwum N-K, Woods G, Velleman Y, Fleming F, Stothard JR. Tailoring water, sanitation, and hygiene (WASH) targets for soil-transmitted helminthiasis and schistosomiasis control. Trends Parasitol. 2018;34(1):53–63. doi: 10.1016/j.pt.2017.09.004 29055522

[13] Kulinkina AV, Kosinski KC, Plummer JD, Durant JL, Bosompem KM, Adjei MN, et al. Indicators of improved water access in the context of schistosomiasis transmission in rural Eastern Region, Ghana. Sci Total Environ. 2017;579:1745–55. doi: 10.1016/j.scitotenv.2016.11.140 27939198 PMC5226254

[14] Armah P, Kicha D. Primary health care in Ghana: The structure and functions in relation to preventing neglected tropical diseases. Archiv Euromed. 2020;10(3):12–7. doi: 10.35630/2199-885x/2020/10/3.2

[15] UN. The Sustainable Development Goals, 2030. 2015 [cited 2026 Mar 12]

[16] WHO. Universal health coverage (UHC), SDG Target 3.8. 2025 [cited 2026 Mar 12]

[17] de Souza DK, Yirenkyi E, Otchere J, Biritwum N-K, Ameme DK, Sackey S, et al. Assessing lymphatic filariasis data quality in endemic communities in ghana, using the neglected tropical diseases data quality assessment tool for preventive chemotherapy. PLoS Negl Trop Dis. 2016;10(3):e0004590. doi: 10.1371/journal.pntd.0004590 27028010 PMC4814091

[18] Adokiya MN, Awoonor-Williams JK, Beiersmann C, Müller O. The integrated disease surveillance and response system in northern Ghana: Challenges to the core and support functions. BMC Health Serv Res. 2015;15(1):288. doi: 10.1186/s12913-015-0960-7 26216356 PMC4515924

[19] Adusei AB, Bour H, Amu H, Afriyie A. Community-based health planning and services programme in Ghana: A systematic review. Front Public Health. 2024;12:1337803. doi: 10.3389/fpubh.2024.1337803 38504682 PMC10948426

[20] Braimah JA, Sano Y, Atuoye KN, Luginaah I. Access to primary health care among women: the role of Ghana’s community-based health planning and services policy. Prim Health Care Res Dev. 2019;20:e82. doi: 10.1017/S1463423619000185 32799990 PMC8060816

[21] Adongo WA, Osei CK, Wongnaa CA. Contribution of nontimber forest products to rural household income in the Kassena-Nankana West district of Ghana. For Prod J. 2019;69(3):217–27. doi: 10.13073/fpj-d-18-00049

[22] Asare KY, Mensah JV, Agyenim JB, Tenkorang EY. Sustainability of alternative livelihood strategies in selected sand mining communities in the Ga South Municipality and Gomoa East District of Ghana. Cogent Soc Sci. 2024;10(1):2340436 doi: 10.1080/23311886.2024.2340436

[23] GSS. Population of Regions and District Report. In: 2021 census reports. Ghana Statistical Service; 2021 [cited 2026 Mar 13].

[24] Akuffo RA, Sanchez C, Amanor I, Amedior JS, Kotey NK, Anto F, et al. Endemic infectious cutaneous ulcers syndrome in the Oti Region of Ghana: Study of cutaneous leishmaniasis, yaws and Haemophilus ducreyi cutaneous ulcers. PLoS One. 2023;18(9):e0292034. doi: 10.1371/journal.pone.0292034 37756291 PMC10529585

[25] Danso F, Appiah MA. Prevalence and associated factors influencing stunting and wasting among children of ages 1 to 5 years in Nkwanta South Municipality, Ghana. Nutrition. 2023;110:111996. doi: 10.1016/j.nut.2023.111996 37003173

[26] Adusei AB, Ayiku RNB, Amarteyfio KANA, Bondzie EPK, Adjei-Banuah NY, Abdul-Samed A-B, et al. Disparities in the access and provision of mental health services as part of primary health care: A case study of Ga-South district in the Greater Accra region. Front Public Health. 2025;13:1537955. doi: 10.3389/fpubh.2025.1537955 40416684 PMC12098029

[27] Koka E, Yeboah-Manu D, Okyere D, Adongo PB, Ahorlu CK. Cultural understanding of wounds, buruli ulcers and their management at the Obom sub-district of the Ga South municipality of the greater Accra region of Ghana. PLoS Negl Trop Dis. 2016;10(7):e0004825. doi: 10.1371/journal.pntd.0004825 27438292 PMC4954709

[28] Ahorlu CK, Koka E, Yeboah-Manu D, Lamptey I, Ampadu E. Enhancing Buruli ulcer control in Ghana through social interventions: A case study from the Obom sub-district. BMC Public Health. 2013;13:59. doi: 10.1186/1471-2458-13-59 23339623 PMC3554508

[29] GSS. Kassena Nankana West district statistical service report. Available from: https://statsghana.gov.gh/gssmain/fileUpload/pressrelease/Kasena%20Nankana%20Municipal.pdf

[30] Okyere D, Ocloo EK, Owusu L, Amoako YA, Tuwor RD, Koka E, et al. Improving experiences of neglected tropical diseases of the skin: Mixed methods formative research for development of a complex intervention in Atwima Mponua District, Ghana. PLOS Glob Public Health. 2024;4(6):e0002833. doi: 10.1371/journal.pgph.0002833 38870111 PMC11175470

[31] Tuwor RD, Mtuy TB, Amoako YA, Owusu L, Oppong MN, Agbanyo A, et al. Stigma experiences, effects and coping among individuals affected by Buruli ulcer and yaws in Ghana. PLoS Negl Trop Dis. 2024;18(4):e0012093. doi: 10.1371/journal.pntd.0012093 38683858 PMC11081504

[32] Phillips RO, Owusu L, Koka E, Ocloo EK, Simpson H, Agbanyo A, et al. Development of an integrated and decentralised skin health strategy to improve experiences of skin neglected tropical diseases and other skin conditions in Atwima Mponua District, Ghana. PLOS Glob Public Health. 2024;4(1):e0002809. doi: 10.1371/journal.pgph.0002809 38241242 PMC10798462

