# Supplementary material for: Integrating neglected tropical diseases into primary healthcare services in Ghana: Results from an initial pilot
Source: PLoS Negl Trop Dis. 2026 Oct 7;20(10):e0014792. doi: 10.1371/journal.pntd.0014792 (PMC13660979; doi:10.1371/journal.pntd.0014792)
Supplement: Supplementary file 1 [file pntd.0014792.s001.docx]

**Comments to Authors**

**Title: Mainstreaming Neglected Tropical Diseases into Primary Healthcare services** in Ghana: results from an initial pilot

**Overall comments**

Overall, the article is well written. This work NTD is a priority and it is good you work on this vital public health concern which is helpful in control and elimination of NTDS.

Active community-based case detection, and linkage to PHC is vital, however unless we work on capacity building at PHCU level it is difficult to address UHC

**Title**

The objective of the study and the methods do not align with the study title

**Abstract**

Line 32- NTDs affects billon individuals globally, not millions

Line 32- the WHO roadmap is 2021-2030, not 2030 road map

Lines 57-58 Why patient compliance with referrals varied with such degree <10%- 92%

Why the compliance is < 10%, what are the factors which contributes to this and how do you address this for future scale up of the interventions

What is the qualification of the Community health officers? are they trained medical personnel?

**Introduction**

Line 105 Do you think leprosy which is under elimination phase in most countries is found in 82% of districts in Ghana?

Line 113- same comment as above, 2021-2030 Road map

**Methods**

In the study setting part, why do you exclude the western and Eastern zones of the country? In order to balance geographical representation

In LMICs, the main gap is availability of standard care package at PHCU or availability of guideline for management of NTDs.

Preparing care package, capacity building of Health care workers, and availing affordable services will enhance quality and access to sevices

Line 181 How about hyper-pigmented lesions, common among skin NTDS especially when the wound is going to heal

Lines 216-219 stated as “The training was held to equip health care workers with the technical know-how to conduct effective piloting of the NTD-integrated home visit register, in aspects of effective home visiting and reporting.”

This implies the home visit and case identification was conducted by health care workers, not community health workers. Thus, how is the feasibly of using the limited health care workforce for hours to house or home visit?

Did any expert or specialists on NTDS was engaged in confirming the diagnosis made by CHOs?

Though your work on early detection and referral within the primary healthcare system can strengthen early detection, surveillance, and linkage to care within an integrated primary healthcare system, unless we work on capacity building of HCWs and availability of basic skin/ wound care supplies at PHCU it is difficult to address UHC. I assume this part is lacking in this pilot intervention.

**Results and Discussions**

In Table 2, Why do you think Nkwanta South has 23.4 % skin lesions, whereas Kassena

Nankana West has 7.3 % skin lesions. However, the visible deformity is 0% in Nkwanta South and 18.4 Kassena Nankana West. Is it due to access or Health facility set-up?

Surprisingly in Ga South the visible deformities are 81.6%. I need justification for these varied disabilities despite all these districts are almost homogenous considering a rural community with similar set up .??

Line 322-325 stated as “In Kassena Nankana West, compliance was excellent, with 92.1% (281/305) of referred patients reportedly visiting a health facility. Conversely, referral compliance was poor in the other two districts, with 52% in Nkwanta South and falling below 10% in Ga South”

Why is such discrepancy, specifically referral compliance is down to 52% or < 10 % in two districts.

Line 357-359 i“ The integration of NTD case finding into the CHPS system represents a paradigm shift from population-level mass drug administration (MDA) toward individualized patient centered care “

This sentence indicates as if this study gives guidance on MDA, I think based on the result of this study we cannot conclude anything about MDA
