# Supplementary material for: Integrating neglected tropical diseases into primary healthcare services in Ghana: Results from an initial pilot
Source: PLoS Negl Trop Dis. 2026 Oct 7;20(10):e0014792. doi: 10.1371/journal.pntd.0014792 (PMC13660979; doi:10.1371/journal.pntd.0014792)
Supplement: Supplementary file 2 [file pntd.0014792.s002.docx]

10th July 2026

**Kumasi Centre for Collaborative Research**

**Kwame Nkrumah University of Science and Technology**

**Kumasi, Ghana**

**The Editor**

**PLoS NTD Journal**

Dear Sir,

Re: ‘Integrating Neglected Tropical Diseases into Primary Healthcare services in Ghana: results from an initial pilot’ PNTD-D-26-00911


My co-authors and I have taken note of the review comments and revised the manuscript as suggested.

We wish to submit the revised manuscript for publication in your esteemed journal. In the attached reply, the reviewer questions are in black font and our responses are in red font.

We look forward to the next steps towards the publication of our manuscript.

Yours Sincerely,

Yaw Ampem Amoako

Corresponding author

**Journal Requirements:**

If the reviewer comments include a recommendation to cite specific previously published works, please review and evaluate these publications to determine whether they are relevant and should be cited. There is no requirement to cite these works unless the editor has indicated otherwise.

1) Thank you for including an Ethics Statement for your study. Please include:
   i) A statement that formal consent was obtained (must state whether verbal/written) OR the reason consent was not obtained (e.g. anonymity). NOTE: If child participants, the statement must declare that formal consent was obtained from the parent/guardian.].

Response: We have indicated that the informed consent was of the written type.

2) Please amend your detailed Financial Disclosure statement. This is published with the article. It must therefore be completed in full sentences and contain the exact wording you wish to be published.

i) State the initials, alongside each funding source, of each author to receive each grant. For example: "This work was supported by the National Institutes of Health (####### to AM; ###### to CJ) and the National Science Foundation (###### to AM).".
If you did not receive any funding for this study, please simply state: u201cThe authors received no specific funding for this work.u201d
Response: The funding statement has been added to the section as follows:

‘The implementation activity was funded on the USAID project Act to End NTD West Consortium with FHI 360 as the Consortium lead and World Vision Ghana as the implementing partner providing technical assistance to the Ghana Health Service. The funders had no role in study design; in the collection, analysis and interpretation of data; in the writing of the report; and in the decision to submit the manuscript for publication.’

**Point-by-point response to reviewers' comments:**

Reviewer #1: The objectives of the study are described in terms of detecting clinical signs of some selected NTDs by means of integrated surveillance system with trained community health officers (CHOs) for subsequent referral and linkage to the nearby health facilities. However, this characterization doesn't imply proper articulation of testable hypothesis. It's not clear as well whether the study design is meant to test the effectiveness of "integrated NTD surveillance" or "mainstreaming of NTDs into the PHC system". Also, it is not clear whether the study settings and health facilities selected were known endemic districts for the 7 target NTDs. Trachoma is eliminated as a public health problem in Ghana several years ago and it's one of the 7 infections/conditions selected for integrated surveillance. There was no explanation about the sample size and the plan for statistical analysis.

Response: We sincerely thank the reviewer for the comment. The primary aim of the study is to evaluate the feasibility and effectiveness of an integrated NTD surveillance system at the community level, rather than broader health system mainstreaming. We have revised the text throughout the manuscript to ensure consistent terminology and to clearly distinguish this from the concept of full integration of NTD services into the PHC system. We have revised the section on study aim and it now reads:

The primary goal of this pilot study was to assess the feasibility of integrating selected NTD-related syndromic indicators into the routine primary healthcare using Community-Based Health Planning and Services (CHPS) home visit register in chosen districts by collaborating with stakeholders at national, sub-national, and district levels. We evaluated identification, referral, and referral-completion processes for suspected cases.’ (line 148-153).

**Description of study setting and endemicity:**
We have now provided additional details regarding the study settings, including justification for site selection. The selected districts are known to be endemic or historically endemic for the targeted NTDs, with ongoing risk of residual or undetected cases. This information has been added to the Study Setting subsection (line 170-171) to give appropriate epidemiological context.

**Inclusion of trachoma in the surveillance package:**
We acknowledge the reviewer’s concern regarding the inclusion of trachoma despite its elimination as a public health problem in Ghana. Trachoma was included as part of the integrated surveillance package to support post-elimination surveillance and to ensure early detection of any potential recrudescence. We have now clarified this rationale in the manuscript (line 110-112).

Regarding the sample size determination and statistical analysis plan, we added the following sentences to address the review concern (line 306-309):

‘The analysis was primarily descriptive and focused on assessing implementation feasibility, uptake, indicator detection, referral patterns, and referral completion. No formal hypothesis-testing comparisons between districts were planned.’

Reviewer #2: The study is framed as an implementation pilot, and its design is broadly appropriate to that purpose: a descriptive, multi-zone deployment embedded in a routine community platform is a reasonable way to test whether CHOs can operationalise integrated NTD syndromic screening. However, the objectives are stated programmatically rather than as a testable hypothesis, with no predefined feasibility criteria or success thresholds (lines 35–38, 140–143), so the findings are narrated post hoc rather than judged against an a priori benchmark. The design is also uncontrolled, with no baseline or non-integrated comparator, and the qualitative monitoring component referred to in the Methods is not actually reported—a notable gap given that the referral-compliance disparity is the headline finding.

The population is described at district level (lines 161–175) but not at the analytic unit level: the within-subdistrict sampling frame is unspecified and, critically, no denominator is defined for the screened population, so the 1,932 visits and 1,117 observations cannot be situated against an eligible base and representativeness cannot be assessed. Sample size and power are not formally addressed—defensible for a feasibility pilot, but several key indicators rest on very small counts (blood in urine n=20; hydrocele n=42) yet are reported with confident percentages and comparative language. The statistical approach (descriptive analysis only) is appropriate in type but is not matched by appropriate caution: proportions are presented without confidence intervals, Table 2 denominators are ambiguous, and comparative claims imply contrasts that were never formally tested. The study is adequately sized to demonstrate feasibility and CHO uptake, but underpowered to support the district-level epidemiological contrasts and zone-attributable explanations advanced in the Discussion.

On ethical and regulatory requirements, the essentials are in place—institutional ethics approval, local permissions, and informed consent are reported (lines 145–151)—but the approval number (CHRPE/AP/335/19) appears to predate the 2023 implementation by several years and should be confirmed as covering this activity. Given household screening for sensitive signs, the authors should also clarify consent procedures for minors and how identifiable referral data were handled. In sum, the methods are fit for the narrow feasibility purpose but are described with insufficient precision and interpreted more strongly than the descriptive, uncontrolled, purposively sampled design can support.

Response: We have now revised the Methods section to clarify that the analytic unit is the individual screened during community-level visits conducted by trained CHOs.

Definition of denominator and representativeness: Given the feasibility nature of the study and the community-based, non-probability screening approach, a true population-level denominator was not formally available. We have now explicitly stated this limitation and clarified that the reported 1,932 visits and 1,117 observations represent programmatic screening outputs rather than a representative sample of the underlying population. Corresponding text has been added to the Methods and Limitations sections (Lines 256-260; 476-489).

Sample size and power considerations: We acknowledge that sample size and statistical power were not formally addressed. As correctly noted by the reviewer, this study was designed as a feasibility/pilot implementation rather than a hypothesis-testing study. We have now explicitly stated that no formal sample size calculation was performed and that the sample reflects operational implementation within the study period. This clarification has been added to the Methods section (Lines 249-250).

Interpretation of small counts: We appreciate the concern regarding the reporting of indicators based on small numbers (e.g., blood in urine, hydrocele). We have revised the Results and Discussion sections to temper the interpretation of these findings, avoiding comparative or definitive language where counts are low. We now explicitly acknowledge that these estimates should be interpreted with caution due to small sample sizes (Lines 476-489)

Presentation of proportions and statistical uncertainty: We have now updated the Results section to include 95% confidence intervals for key proportions where appropriate (Table 3). In addition, we have revised the language throughout to ensure consistency with a descriptive analytical approach and to avoid overinterpretation of the findings.

Reviewer #3: -The objectives of the study clearly articulated with a clear testable hypothesis stated but do not align with the study title
-In the study design it would have been better if qualitative component included to determine enablers and barriers
-The population clearly described and appropriate for the hypothesis being tested
Repetitiveness is lacking, the western and Eastern part of the country has not been represented and also proper justification has not been provided
IRB clearance and informed consent procedures has been met.

Response: We appreciate the acknowledgment that the study population is clearly described and appropriate for the hypothesis being tested, and that ethical requirements, including IRB clearance and informed consent procedures, were adequately met.

The current study population was primarily drawn from specified study districts drawn from the 3 ecological zones of Ghana. This was largely due to logistical constraints and the availability of collaborating centres during the study period. We acknowledge that this may affect the generalizability of our findings.

**Results**

Reviewer #1: The analysis lacks depth and clarity. The authors presented the results of the integrated surveillance with the proxy indicators they outlined for the CHOs. Not clear whether the 7 target NTDs were clearly described for the CHOs. The results were discussed as clinical signs presented by visible indications (observation) without additional investigations by means of interviews or other simple diagnostic means (using recognition cards, etc). The figures and tables used are too shallow and do not represent substantive evidence and statistical data.

Response: We thank the reviewer for these important observations. We have clarified in the Methods section that CHOs were trained using standardized case definitions, visual aids, and practical exercises to recognize the seven selected NTD-related indicators (line 239-240). The intent of the pilot was not to establish definitive diagnoses but rather to assess the feasibility of community-level identification of syndromic indicators that warrant referral for further assessment.

We have revised the Results and Discussion sections to consistently describe findings as syndromic indicators rather than confirmed disease outcomes. Additional text has been added to emphasize that the observed signs were preliminary screening signals captured during routine household visits and were not intended to replace diagnostic confirmation.

To improve data presentation, we have revised tables and figures to include clearer denominators, counts alongside percentages, and measures 95% CI where appropriate (Table 3). We have also expanded the narrative accompanying the results to better explain the interpretation and limitations of the findings.

Reviewer #2: The results are broadly consistent with the stated descriptive analysis plan—frequencies of the seven indicators by district, referral proportions, and implementation rates—so there is no evidence of analyses beyond what the Methods described. The limitation is the reverse: the descriptive plan is executed with comparative and quasi-inferential language ("most prevalent," "marked geographical variation," lines 293–302) that implies contrasts never formally tested. The analysis as planned is appropriate; the interpretation layered onto it is not fully matched by the plan.

The results are not yet clearly or completely presented, and this is the most consequential weakness. Percentages are reported against ambiguous denominators—Table 2 cell values cannot be reconciled to a single base (column total, district row total, or screened population), so the same figure carries different meanings depending on the reader's assumption (e.g., Nkwanta South skin lesions are shown as 23.4% against n=651 but represent 214/248 of that district's recorded indicators). Several in-text values do not match the tables or figures, and the visit and referral denominators (672, 409, 851; lines 316–318) do not align cleanly with the facility structure described earlier or with the 1,932 total. Key quantities also rest on very small counts reported without uncertainty (blood in urine n=20; hydrocele n=42), and a single derived metric central to the referral pathway—the positive predictive value of CHO identification—is referred to but never presented. Completeness requires counts alongside every percentage, a single labelled denominator per table column, exact text–table–figure correspondence, and 95% confidence intervals for the headline proportions.

The figures and tables are adequate in concept but not in execution. Table 1 is essentially a facility inventory that could be condensed or moved to a supplement. Table 2 needs explicit denominators in each column header to be interpretable. Figures 3–5 lack value labels and confidence intervals, and their axis counts only approximate the in-text numbers; Figure 4's legend ("Facility visits from referrals" vs "Home Referrals to health facility") is ambiguous as to which bar is numerator and which is denominator, and the trained-worker denominators differ between Methods (64 CHOs) and Results (87 workers), leaving Figure 5's base unclear. None of these are quality problems that require new data—they are matters of labelling, reconciliation, and the addition of denominators and uncertainty—but they must be resolved before the descriptive findings can be independently verified.

Response: In response to the review comment, we have re-organised Table 2 to include column totals/ denominators as suggested. We also corrected the computational errors identified in the percentages.

Further, we have also opted to present the information in Figures 3 and 4 in tabular form (Table 3) for easier understanding and provided 95% CI for ‘Proportion of home visits leading to referral’ and ‘Proportion of referral resulting in actual attendance to health facilities.

Reviewer #3: the results are clearly presented and the tables/figures have suffient quality

Response: We thank the reviewer for the positive assessment of the presentation of the results and the quality of the figures and tables. We have nevertheless implemented additional revisions suggested by the other reviewers to further improve clarity, consistency, and interpretability of the results.

**Conclusions**

Reviewer #1: The conclusions are as good as the results discussed above. Nothing substantive as it pertains to mainstreaming of NTDs but something about integrated surveillance and referral linkages.

Response: We thank the reviewer for this observation. We have revised the Discussion and Conclusion sections to focus on the specific findings generated by the pilot, namely the feasibility of integrating NTD-related syndromic surveillance into routine CHPS activities, successful uptake by CHOs, and the identification of a referral-compliance gap.

We have moderated statements implying broader health-system mainstreaming and now clearly distinguish between integrated surveillance, which was evaluated in this pilot, and broader NTD service integration, which remains a longer-term objective.

Reviewer #2: The conclusions are only partially supported by the data presented. The core claim—that integrating NTD syndromic indicators into the CHPS home-visit register is operationally feasible and is taken up by CHOs (lines 422–428)—is reasonably supported by the high implementation rate and the volume of indicator capture. The identification of a referral-completion bottleneck is likewise a fair reading of the compliance disparity. However, several conclusions extend beyond the evidence: the characterisation of the approach as "a paradigm shift from population-level MDA toward individualized patient-centered care" (lines 357–359), the readiness for "phased national roll-out" (lines 405–406), and the disease-specific epidemiological patterns inferred from non-specific proxy signs all outpace what a short, uncontrolled, purposively sampled descriptive pilot can demonstrate. No data on case confirmation, treatment, morbidity outcomes, or cost were generated, so the implied health and efficiency benefits are hypothesised rather than shown. The conclusions need to distinguish explicitly what was demonstrated (feasibility of integrated data capture, high CHO uptake, a referral gap) from what is hoped (scalability, health impact, the efficacy of proposed remedies).

The limitations are described, and the authors deserve credit for candour: they acknowledge the short three-month window, the reliance on suspected cases without confirmatory diagnostics, the absence of a qualitative component to explain referral behaviour, and the inability to assess long-term sustainability (lines 394–403). This is a genuine strength relative to many implementation reports. The limitations section is nonetheless incomplete in the areas that most affect validity—it does not address the undefined population denominator, the ambiguous percentage bases, the small cell counts underlying several headline figures, or the misclassification risk inherent in mapping single syndromic signs to specific diseases. Folding these into the limitations would materially strengthen the manuscript's internal consistency.

The authors do engage with how the data advance understanding, and they do so credibly within the feasibility frame: the work shows that an existing routine community platform can be repurposed for integrated NTD surveillance, that frontline uptake is achievable, and that detection alone is insufficient without active linkage to care. This is a useful contribution to the operational evidence base, and the candid surfacing of the referral-compliance gap is its most transferable insight. The advance would be stronger still if the discussion separated demonstrated feasibility from the broader inferential and policy claims, and framed the district-level explanations as hypotheses for prospective testing.

Public health relevance is clearly and appropriately addressed. The manuscript situates the work within the WHO 2030 NTD roadmap, the UHC agenda, and Ghana's NTD master plan (lines 108–123, 341–353), and it draws actionable programmatic implications—patient navigation, transport subsidies, audit-and-feedback learning from the high-performing district (lines 405–413). These are sensible and relevant, provided they are presented as plausible, untested options rather than validated solutions. On balance, the conclusions are relevant and partly supported, but require moderation to align with the descriptive design, and the limitations require modest expansion to cover the denominator, classification, and uncertainty issues.

Response: we have revised the Discussion and Conclusion sections to clearly separate demonstrated findings from future opportunities and hypotheses. We have removed or substantially moderated statements regarding paradigm shifts in care delivery, national scale-up readiness, and disease-specific epidemiological interpretations based solely on proxy indicators.

The revised conclusion now emphasizes three findings supported by the data: (i) the operational feasibility of integrating NTD syndromic indicators into the CHPS home visit platform, (ii) strong uptake by trained CHOs, and (iii) identification of important gaps in referral completion. We have also expanded the limitations section to include discussion of undefined population denominators, potential misclassification arising from syndromic indicators, small cell counts for some observations, and the descriptive nature of the study design.

Reviewer #3: the conclusion supports the data presented but in some it doesn't . Example The recommendation about MDA is not supported by study data and do not align with the study objectives

The public health relevance of the study has been clearly stated

Response: We thank the reviewer for this important observation. Our conclusions focused mainly on findings supported by the pilot data, specifically the feasibility of integrated surveillance and referral processes within the CHPS system.

**Editorial and Data Presentation Modifications?**

Reviewer #1: Major revisions required.

Reviewer #2: Minor Comments

Abstract
• The Abstract reports “67% compliance” overall (line 52) but the Results give district-specific compliance ranging from <10% to 92.1% (lines 322–325); an aggregate figure masking this range is misleading and should be removed or contextualised.

Response: The abstract has been revised to present referral compliance in a more balanced manner, highlighting the substantial variation observed across districts rather than relying solely on the aggregate value.
• State the study design explicitly as a three-district descriptive implementation pilot, and avoid the disease-specific attributions (“suggestive of schistosomiasis”, line 54) in favour of syndromic wording.

Response: Our methods clearly mentioned the study as a pilot implementation in 3 districts (line 40-41). We have deleted the disease specific attribution ‘suggestive of schistosomiasis’.

• Clarify whether “1,117 suspected cases” (line 51) refers to individuals or to indicator observations, since an individual may present more than one sign.
Introduction

Response: The abstract and Results sections have been revised to clarify that the 1,117 records represent observations of NTD-related indicators identified during home visits. Some individuals could potentially present with more than one indicator (line 52 and 325-326)
• The count of endemic NTDs is stated as 21 globally (line 90) and 14 in Ghana (line 98); confirm against the current WHO list (now 21 conditions/disease groups) and ensure consistency with the GHS NTD Master Plan reference [9].

Response: We indicated current WHO classification list 21 conditions as NTDs (line 89) indicates 14 of these are endemic in Ghana as per the Ghana NTD Master Plan (line 97).
• The claim that NTDs are “entirely preventable” (line 96) is an overstatement for several conditions; soften to “preventable and controllable.”

Response: The word ‘entirely’ has been deleted from the section and replaced with ‘largely’. (line 95)
• Reference [9] is dated 2021–2025; verify it remains the current master plan or note the successor document.

Response: Although the NTD Masterplan for Ghana is under review, the reference cited remains the available published master plan for Ghana at the time of writing this manuscript.

Methods
• Specify the sampling frame within each subdistrict (how households and individuals were selected for the home visit) and whether all household members were screened (lines 155–169).

Response: This pilot was embedded within routine Community-based Health Planning and Services (CHPS) activities and therefore did not employ probability-based household sampling. Rather, Community Health Officers (CHOs) conducted household visits within their assigned CHPS catchment areas according to their routine outreach schedules and house-to-house visitation plans. We have revised the methods to address the review concern by adding the following sentences (line 237-247):

‘Household visits were conducted as part of routine outreach activities by CHOs within their assigned CHPS catchment areas. No predefined population sampling frame or population-level denominator was established because the pilot was designed to evaluate operational feasibility rather than estimate disease prevalence. During their household visits, CHOs screened available household members using the revised register and recorded the presence or absence of the predefined NTD-related indicators as captured in the new register. During each household visit, all available household members who were present at the time of the visit and consented to participate were screened for the seven NTD indicators. Individuals who were absent during the visit were not assessed unless encountered during subsequent routine visits.’

• The operational definition of hydrocele as “any scrotal swelling” (line 182) and lymphoedema as “swollen limbs” (line 187) are very broad; note expected specificity limitations.

Response: The operational definition of suspected hydrocele and lymphoedema prioritised sensitivity over specificity and may have included conditions unrelated to hydrocele or lymphoedema. We have acknowledged the limited specificity of these proxy indicators and expanded the Discussion and Limitations sections to highlight the possibility of alternative causes and potential misclassification.

We acknowledge that the operational definition of suspected hydrocele as any scrotal swelling prioritised sensitivity over specificity and may have included conditions unrelated to lymphatic filariasis. Similarly, swollen limbs may result from several conditions, including cellulitis, podoconiosis, venous disease, trauma, and therefore should be interpreted as a syndromic screening indicator rather than a disease-specific diagnosis.
• State how referral compliance was ascertained (facility register linkage, CHO follow-up, or patient self-report) and the follow-up window (lines 312–325).

Response: We have addressed the review concern by adding the following sentences to the methods section (line 250-253):

‘Referral compliance was assessed through routine follow-up conducted by CHOs and district monitoring teams using referral records and documented evidence of attendance at the designated health facility within the implementation period.’

• Indicate whether the electronic and paper-based registers produced concordant data, given both were piloted (lines 137–138).

Response: We have clarified in the results section (line 388-390) that ‘Review of both the electronic and paper registers used in parallel during the piloting showed that no major discrepancies affecting interpretation were identified during the monitoring activities.’

• Software is listed as GraphPad Prism 6.0 and Stata 12 (lines 272–274); for a descriptive analysis, clarify what each tool was used for, and note that these are dated versions.

Response: Done as suggested
• The ethics approval number (CHRPE/AP/335/19; line 149) predates the 2023 implementation by several years—confirm the approval covers this activity or cite an amendment.

Response: We confirm that the cited ethics approval covered the implementation activity.

Results
• Add the denominator to every Table 2 column header and reconcile the percentage base (column vs row); the current presentation is ambiguous (lines 309–310).

Response: Table 2 has been revised as suggested
• Report counts with all percentages in the text (e.g., eye complications “~70%” and lymphoedema “81%” in KNW; lines 300–302).

Response: Done as suggested (line 334-336)
• Figures 3–5 lack value labels and confidence intervals; axis counts should match in-text numbers exactly.

Response: The figures 3 and 4 have been represented as Tables to improve clarity.
• Fig 4 legend labels (“Facility visits from referrals” vs “Home Referrals to health facility”) are confusing—clarify which bar is numerator and which is denominator.

Response: Figure 4 has now been deleted
• The PPV of CHO identification is not reported despite confirmation being central to the referral pathway; if facility confirmation data exist, present them.

Response: The indicators used in this pilot were intentionally designed as syndromic screening tools and should not be interpreted as confirmed diagnoses. Several indicators have important differential diagnoses that would require clinical assessment and diagnostic confirmation at health facilities. Thus, were are unable to report the PPV of CHOs since we did not include data on diagnostic confirmation.
• The training/implementation denominators differ between the Methods (64 CHOs trained; line 220) and Results (87 workers trained; line 329)—reconcile and define which group Fig 5 describes.

Response: in line 228-232, we stated as follows:

‘A total of 64 Community Health Officers (Kassena Nankana =21; Ga South =25; Nkwanta South = 18) were trained. In addition, 23 Disease Control officers, Public Health nurses and Health information officers from the districts were trained to monitor and provide supervision oversight during implementation.’ Thus the 87 refers to the total number of all workers trained (and not CHOs only).

Discussion
• Avoid “validating the ability of CHOs to act as effective surveillance sentinels” (lines 350–351); the pilot did not validate accuracy against a reference standard.

Response: We have revised the section by deleting the word ‘validating’. The section now reads:

‘The data collected by Community Health Officers (CHOs) revealed distinct epidemiological patterns - such as the high prevalence of blood in urine in the coastal Ga South district and lymphoedema in the northern Kassena Nankana West district - demonstrating the ability of CHOs to act as effective surveillance sentinels to identify and report syndromic indicators.’ (line 399-403)
• The reverse-causality and confounding caveats applied to district differences should be stated explicitly rather than implied.

Response: We have revised the Discussion to clearly present district-level explanations as hypotheses rather than demonstrated causal mechanisms. We now explicitly state that these factors require further investigation.
• The limitations paragraph (lines 394–403) is appropriate but should be expanded to include the denominator and misclassification issues raised in the major comments.

Response: We acknowledged in the limitations section issues relating to syndromic classification (line 475-476), absence of confirmatory diagnostics (line 428). We have expanded the section to include the descriptive nature of the study design and lack of population denominators, and small sample sizes for some indicators (line 478-480).
• Temper “paradigm shift” (line 357) and “dismally low” (line 365) toward neutral scientific register.

Response: We have revised the sections as requested
Language and Presentation
• Standardise terminology: “lymphoedema” vs “lymphedema,” “sub-national” vs “subnational,” and “Kassena Nankana” vs “Kassena-Nanka” (Table 1 header, line 288).

Response: The sections have been revised appropriately
• Several sentences are incomplete or have agreement errors (e.g., “The recorded NTD related data as captured…”, lines 228–229); a careful proofread is needed.

Response: The section has been revised as suggested
• Define all abbreviations at first use (ICCC, CHIM, MMDP) and ensure consistent expansion.

Response: All abbreviations were defined before their first use.
• Ensure figure callouts (Fig 1–Fig 5) appear in numerical order and that all figures are cited in the text.

Response: Figures 3 and 4 have been deleted. The original figure 5 is now figure 3.
• Check reference formatting and access dates (several references list future-dated access notes, e.g., “12th March, 2026”).

Response:

**Summary and General Comments**
Reviewer #1: The weaknesses detailed above needs to be addressed.

Response: We have revised the manuscript to address all the review comments.

Reviewer #2: This manuscript reports a three-district implementation pilot integrating seven syndromic NTD indicators into Ghana’s Community-based Health Planning and Services (CHPS) home-visit register, with Community Health Officers (CHOs) conducting screening during routine household visits over a three-month period. The study is programmatically timely and well aligned with the WHO 2030 NTD roadmap and the UHC agenda, and it addresses a genuine operational question: whether routine community platforms can be leveraged for integrated NTD case finding and referral. The engagement of national, sub-national and district stakeholders, the use of an existing routine data tool, and the explicit attention to the referral pathway are real strengths. However, several issues require substantial attention before the manuscript can support its stated conclusions. The numerical results are internally inconsistent and the denominators underlying the reported percentages are ambiguous, which currently prevents independent verification of the descriptive findings. The proxy indicators are treated, in places, as disease-specific despite acknowledged non-specificity, risking overstatement of disease burden. The analysis is wholly descriptive with no measures of uncertainty, yet comparative and quasi-causal language is used throughout, and the policy and generalisability claims exceed what a short, purposively sampled, uncontrolled pilot can demonstrate. For these reasons I recommend a Major Revision. With transparent denominators, reconciled figures, syndromic reframing, added uncertainty estimates, and proportionate conclusions, the manuscript could make a meaningful contribution to the evidence base on integrating NTD surveillance into primary healthcare.

Major Comments

1. The study is positioned as assessing the feasibility of integrating NTD indicators into the CHPS home-visit register for national scale-up (Abstract; lines 35–38, 140–143). However, the analysis reports outputs (visits conducted, signs recorded, referrals made) without a clear denominator describing the eligible population or the expected versus achieved visit volume. The reported totals are internally inconsistent: the Abstract and Results state 1,932 home visits (lines 51, 291), yet the district figures used for referral rates sum to 1,932 only when expressed as 672 (Ga South), 409 (Nkwanta South) and 851 (Kassena Nankana West) (lines 313–318), while the facility-level visit counts implied elsewhere differ. Without a defined denominator—target households, registered population, or planned visits per CHO over the three months—the reader cannot judge whether case detection reflects genuine feasibility or simply variable field effort across districts. The authors should define the catchment denominator explicitly, report visits per CHO and per facility, and clarify whether 1,932 represents all visits or only visits in which at least one indicator was assessed. This is central to the feasibility claim and to any extrapolation toward scale-up.

Response: We have revised the Methods and Results sections to explicitly define the population under surveillance and clarify the visit counts reported. Specifically, we stated the number of participating CHPS zones, health facilities, Community Health Officers (CHOs), and the catchment population covered by the pilot.

We have also clarified that the 1,932 reported visits represent all home visits conducted during the three-month implementation period in which the revised CHPS register was used and NTD indicators were assessed and recorded, rather than only visits in which a suspected case was identified.

The primary objective of this pilot was to assess the operational feasibility of integrating NTD indicators into routine CHPS activities, including register use, indicator recording, and referral generation, rather than to estimate population-level disease burden or coverage.

We also carefully reviewed the figures reported throughout the manuscript and corrected inconsistencies where identified. The district-specific visit totals (Ga South = 672, Nkwanta South = 409, and Kassena Nankana West = 851) sum to 1,932 and represent the complete set of home visits undertaken during the pilot period. We have revised the text to ensure consistency across the Abstract, Results, tables, and supplementary materials.

We have tempered statements regarding scale-up and highlighted that future implementation studies should include coverage indicators, such as the proportion of eligible households reached, expected versus completed visits, and workload-adjusted performance measures, to better assess effectiveness and scalability.

2. The seven indicators are explicitly described as proxy clinical signs (lines 179–188) and the Discussion acknowledges that cases are “suspected” without confirmatory diagnostics (lines 396–398). However, the manuscript repeatedly maps single signs to specific diseases—for example, blood in urine to urogenital schistosomiasis (lines 250, 306–308) and lymphoedema to lymphatic filariasis—without addressing the well-documented non-specificity of these signs. Self-reported haematuria has limited positive predictive value outside known schistosomiasis transmission foci, and lymphoedema in this setting may reflect non-filarial causes including podoconiosis, venous disease, or trauma. Hydrocele is operationally defined as “any scrotal swelling” (line 182), which will capture numerous non-LF aetiologies. The current framing risks overstating disease-specific burden from what are, at best, syndromic screening signals. The authors should (a) reframe results as syndromic indicator frequencies rather than disease-specific prevalence, (b) explicitly discuss differential diagnoses for each indicator, and (c) quantify, where facility confirmation data exist, the proportion of referred suspected cases subsequently confirmed. This directly affects the validity of the geographical “epidemiological patterns” emphasised in the Discussion (lines 347–353).
Response: We have indicated in the methods that the indicators employed in this study were intended as syndromic screening tools to identify individuals warranting further clinical evaluation rather than to establish disease-specific diagnoses. Consequently, the frequencies reported represent the occurrence of suspected clinical indicators. The chosen indicators prioritized sensitivity over specificity, and this was acknowledged in the limitations section. We have also revised the discussion section on geographical patterns to avoid implying disease-specific epidemiological distributions. These findings are now framed as geographical variation in the distribution of syndromic indicators detected through community screening, which require confirmation through clinical evaluation and diagnostic testing before conclusions can be drawn regarding disease burden. These limitations have been explicitly acknowledged.

3. Several numerical statements cannot be reconciled across the text, tables and figures, which undermines confidence in the descriptive findings. The text states skin lesions were most frequent in Ga South at 59% (386/651) followed by Nkwanta South at “approximately 25%” (lines 294–296), but Table 2 reports the Nkwanta South skin-lesion cell as 214 (23.4%) against a column total of n=651, while the district row total is n=248—so 214/248 (86%) of recorded indicators in Nkwanta South were skin lesions, a very different quantity from the 23.4% shown. The percentage bases in Table 2 are therefore ambiguous (column-wise vs row-wise vs district-population-wise), and the same ambiguity affects every cell. Similarly, the referral denominators in the text (672, 409, 851; lines 316–318) do not match the facility/visit structure described earlier, and Fig 3/Fig 4 axis values approximate but do not exactly correspond to the in-text counts. The authors must standardise all percentages to a single, clearly stated denominator, add the denominator to each table column header, and ensure exact correspondence between text, tables and figures. As presented, the central descriptive results are not independently verifiable.
Response: See response to review comments above. Also see Table 2 and Table 3. The original figures 3 and 4 have now been replaced with Table 3.
4. The referral-compliance disparity is the manuscript’s headline finding (Abstract lines 57–59; Discussion lines 363–371) and is genuinely important. However, the interpretation extends beyond what a three-month, descriptive, uncontrolled pilot can support. Compliance is reported as 92.1% in Kassena Nankana West, 52% in Nkwanta South and <10% in Ga South (lines 322–325), and these differences are attributed to rurality, trust, alternative providers and transport (lines 374–381). These are plausible hypotheses, not demonstrated mechanisms, because the study collected no patient-level data on reasons for non-attendance (a limitation the authors themselves note, lines 398–401). Moreover, “compliance” appears to be ascertained from whether referred patients were recorded as attending—the verification source, time window for attendance, and handling of patients lost to follow-up are not described. The authors should specify how facility attendance was ascertained and over what period, present the compliance denominator transparently, and explicitly frame the district-level explanations as hypotheses to be tested rather than findings. Causal language linking setting to compliance should be moderated throughout.
Response: We have revised the manuscript to (i) clearly describe how referral compliance was measured (line 253-258), (ii) specify the source of attendance verification and follow-up period (line 256); and (iii) moderated our interpretation of district-level differences. We now explicitly present explanations as hypotheses rather than demonstrated determinants.

Referral compliance was defined as the proportion of referred individuals who had documented evidence of attending the designated health facility within the study follow-up period. The denominator comprised all individuals referred from community screening, while the numerator included those with verified facility attendance records. Individuals without documented attendance were classified as non-compliant for the purposes of this analysis. These details have now been added to the Methods section (line 253-258).

We also agree that the study design does not permit causal inference regarding factors influencing referral completion. As noted by the reviewer, no patient-level data were collected on reasons for non-attendance, and therefore the study cannot determine whether the observed differences were attributable to healthcare access, transportation, socioeconomic factors, health-seeking behaviour, trust in health services, or other contextual influences. Consequently, causal language has been removed throughout the manuscript and replaced with more cautious wording indicating that these factors may have contributed to the observed variation and should be investigated in future studies.

5. The statistical approach is limited to descriptive analysis of demographic and clinical variables (lines 274–275), which is reasonable for a pilot but is not matched by appropriate caution in reporting. No confidence intervals, no denominators-with-uncertainty, and no formal comparison accompany the district contrasts, yet the text uses comparative language (“most prevalent,” “marked geographical variation,” lines 293–302) that implies inferential contrast. Given small cell counts—e.g., blood in urine n=20 total, with 15/20 in Ga South (lines 306–308), and hydrocele n=42—point estimates are highly unstable and percentages such as “75%” rest on fewer than 20 observations. The manuscript should (a) report counts alongside every percentage, (b) add 95% confidence intervals (e.g., Wilson intervals) for key proportions, (c) avoid superlative comparative claims where cell counts are small, and (d) state explicitly that observed geographical differences are descriptive and may partly reflect differential CHO effort, training intensity, or recording practice rather than true epidemiological gradients.
Response: We have now provided 95% confidence intervals (Table 3) and moderated the language in the results and discussions as described above and in the manuscript text.
6. The selection of three districts across northern, middle and southern zones is presented as enhancing transferability “across diverse ecological and socio-cultural contexts” (lines 372–374), and the model is described as offering “a valuable template for other countries in sub-Saharan Africa” (lines 385–387). With one district per zone and purposive selection on the basis of NTD endemicity (lines 157–160), the design cannot support claims of representativeness within Ghana, let alone regional generalisability. District-level performance differences (e.g., 100% implementation in KNW vs lower elsewhere) are equally consistent with idiosyncratic local factors—supervision quality, individual CHO motivation, facility density—as with zone-level ecology. The authors should temper generalisability claims, frame the three districts as illustrative case settings rather than a representative sample, and explicitly acknowledge that with n=3 districts the study is underpowered to attribute outcomes to ecological zone.
Response: We have tempered the discussions appropriately to address the review concerns. Further, we have added appropriate sentences to the limitations section to address the concerns
7. Several conclusions are stronger than the descriptive, short-duration design warrants. The Discussion describes the integration as “a paradigm shift from population-level mass drug administration (MDA) toward individualized patient-centered care” (lines 357–359) and recommends phased national roll-out (lines 405–406). While the pilot supports the narrower claim that indicator capture is operationally feasible and acceptable to CHOs, it does not demonstrate improved case confirmation, treatment, morbidity outcomes, or cost-effectiveness—and the authors acknowledge no long-term sustainability data (lines 401–403). Recommendations such as patient-navigation systems and transport subsidies (lines 408–411) are sensible but are presented as solutions when no data on their effect were generated here. The conclusions should be reframed to distinguish what was demonstrated (feasibility of integrated syndromic data capture; high CHO uptake; a referral-completion bottleneck) from what is hypothesised (scalability, health-impact, and the efficacy of proposed remedial interventions). A cost or health-outcome claim should not be implied without supporting data.

Response: We have revised the discussions and conclusions, and we believe the review concerns are addressed by the revisions to the manuscript.

Reviewer #3: The strength of the study has determined encouraging mechanisms for active community based case detection of NTDs
The limitation is a very low referral linkage , even in one of the study districts it was less than 10 %. One of the reasons may be sub-standard quality and access of services at PHCU, which should be part of the intervention

Response: Many thanks for the overall positive feedback. We have revised the manuscript to address all review concerns.

**Comments to Authors (in attached word document)**

**Title: Mainstreaming Neglected Tropical Diseases into Primary Healthcare services** in Ghana: results from an initial pilot

**Overall comments**

Overall, the article is well written. This work NTD is a priority and it is good you work on this vital public health concern which is helpful in control and elimination of NTDS.

Active community-based case detection, and linkage to PHC is vital, however unless we work on capacity building at PHCU level it is difficult to address UHC

**Title**

The objective of the study and the methods do not align with the study title

Response: To better reflect the actual intervention evaluated, we have revised the title to emphasize integrated NTD surveillance within primary healthcare rather than broader NTD mainstreaming and now reads:

‘Integrating Neglected Tropical Diseases into Primary Healthcare services in Ghana: results from an initial pilot’

**Abstract**

Line 32- NTDs affects billon individuals globally, not millions

Response: The correction has been done. (line 32)

Line 32- the WHO roadmap is 2021-2030, not 2030 road map

Response: The section has been revised as suggested (line 35)

Lines 57-58 Why patient compliance with referrals varied with such degree <10%- 92%. Why the compliance is < 10%, what are the factors which contributes to this and how do you address this for future scale up of the interventions

Response: This has been done (line 430-440) and reads:

‘Referral compliance varied markedly across districts, ranging from 92.1% in Kassena Nankana West to 52% in Nkwanta South and less than 10% in Ga South. These differences may reflect variations in healthcare access, transportation barriers, socioeconomic factors, community awareness, stigma, health-seeking behaviour, and the effectiveness of referral follow-up systems. While Kassena Nankana West may have benefited from stronger community–facility linkages and patient follow-up, barriers to linkage-to-care may have contributed to the lower compliance observed in Nkwanta South and Ga South. As our study did not investigate determinants of referral completion, further research is needed to identify district-specific factors associated with referral compliance.’

What is the qualification of the Community health officers? are they trained medical personnel?

Response: We have added a description of CHOs to the Methods section. CHOs are formally trained frontline health professionals employed by the Ghana Health Service and are responsible for delivering community-based primary healthcare services through the CHPS programme. (line 138-141)

**Introduction**

Line 105 Do you think leprosy which is under elimination phase in most countries is found in 82% of districts in Ghana?

Response: Yes, that figure is consistent with reports from Ghana's leprosy control program. Although Ghana achieved the WHO elimination target for leprosy as a public health problem (less than 1 case per 10,000 population) years ago, the disease has not been completely eradicated. New cases continue to be detected annually. A recent evaluation reported that Ghana recorded 1,614 leprosy cases between 2017 and 2022, indicating ongoing transmission in some areas (<https://afenet-journal.org/10-37432-jieph-confpro7-0010/>). We have however chose to delete the 82% figure and replaced it with the word ‘several’.

Line 113- same comment as above, 2021-2030 Road map

Response: Done as suggested.

**Methods**

In the study setting part, why do you exclude the western and Eastern zones of the country? In order to balance geographical representation

Response: Ghana has 3 ecological zones, and this was reflected in the selection of study sites. Furthermore, the choice of districts was intended to provide illustrative implementation settings rather than a nationally representative sample. (line 174-175)

In LMICs, the main gap is availability of standard care package at PHCU or availability of guideline for management of NTDs.

Preparing care package, capacity building of Health care workers, and availing affordable services will enhance quality and access to services

Response: While we agree these to be important, they were nit the focus of our current study.

Line 181 How about hyper-pigmented lesions, common among skin NTDS especially when the wound is going to heal

Response: The definition of skin lesion used was ‘*Skin lesions: hypopigmented or red patches, nodules and skin infiltrations’* The hyperpigmented lesions were captured among the ‘red patches’

Lines 216-219 stated as “The training was held to equip health care workers with the technical know-how to conduct effective piloting of the NTD-integrated home visit register, in aspects of effective home visiting and reporting.”

This implies the home visit and case identification was conducted by health care workers, not community health workers. Thus, how is the feasibly of using the limited health care workforce for hours to house or home visit?

Response: Various health care workers were trained. These included the CHOs, Disease Control Officers, Public Health Officers and Health Information officers. However, only the CHOs participated in the home visits. The numbers of the various cadres of health worjkers trained is indicated in line 235-239 which reads as follows:

‘A total of 64 Community Health Officers (Kassena Nankana =21; Ga South =25; Nkwanta South = 18) were trained. In addition, 23 Disease Control officers, Public Health nurses and Health information officers from the districts were trained to monitor and provide supervision oversight during implementation.’

Did any expert or specialists on NTDS was engaged in confirming the diagnosis made by CHOs?

Response: No specialist diagnostic confirmation was incorporated into the pilot as the objective was to evaluate community-based syndromic screening and referral. We have clarified this in the Methods and acknowledged it as a limitation.

Though your work on early detection and referral within the primary healthcare system can strengthen early detection, surveillance, and linkage to care within an integrated primary healthcare system, unless we work on capacity building of HCWs and availability of basic skin/ wound care supplies at PHCU it is difficult to address UHC. I assume this part is lacking in this pilot intervention.

Response: We do agree with the review comment. However, the current pilot focused primarily on community-level identification, reporting, and referral. Strengthening facility-level capacity, availability of clinical guidelines, training of healthcare workers, and access to essential diagnostic and treatment services are critical next steps for achieving broader NTD integration and Universal Health Coverage. This recommendation has been added to the Discussion section (line 415-418).

**Results and Discussions**

In Table 2, Why do you think Nkwanta South has 23.4 % skin lesions, whereas Kassena Nankana West has 7.3 % skin lesions. However, the visible deformity is 0% in Nkwanta South and 18.4 Kassena Nankana West. Is it due to access or Health facility set-up?

Surprisingly in Ga South the visible deformities are 81.6%. I need justification for these varied disabilities despite all these districts are almost homogenous considering a rural community with similar set up .??

Response: Thank you for this observation. The apparent discrepancy between the proportion of skin lesion cases and visible deformities across the two districts may be explained by differences in case detection patterns, health-seeking behaviour, and access to early diagnosis and treatment. We have added the following sentences to the discussion section to address the review comment (line 417-427):

‘Substantial variation was observed in the proportions of skin lesions and visible deformities across districts. While Nkwanta South reported a higher proportion of skin lesions without visible deformities, Kassena Nankana West and Ga South recorded higher proportions of visible deformities. This pattern may reflect differences in the timing of case detection, health-seeking behaviour, community awareness, referral pathways, or local case-finding activities. In particular, the high proportion of visible deformities in some districts may be influenced by the small number of reported cases, resulting in unstable percentages. Since information on healthcare access, health facility capacity, and diagnostic delays was not assessed, the underlying causes of these district-level differences could not be determined and warrant further investigation.’

Line 322-325 stated as “In Kassena Nankana West, compliance was excellent, with 92.1% (281/305) of referred patients reportedly visiting a health facility. Conversely, referral compliance was poor in the other two districts, with 52% in Nkwanta South and falling below 10% in Ga South”

Why is such discrepancy, specifically referral compliance is down to 52% or < 10 % in two districts.

Response: We have addressed the review concern by adding the following sentences to the discussion section (line 430-440):

‘Referral compliance varied markedly across districts, ranging from 92.1% in Kassena Nankana West to 52% in Nkwanta South and less than 10% in Ga South. These differences may reflect variations in healthcare access, transportation barriers, socioeconomic factors, community awareness, stigma, health-seeking behaviour, and the effectiveness of referral follow-up systems. While Kassena Nankana West may have benefited from stronger community–facility linkages and patient follow-up, barriers to linkage-to-care may have contributed to the lower compliance observed in Nkwanta South and Ga South. As our study did not investigate determinants of referral completion, further research is needed to identify district-specific factors associated with referral compliance.’

Line 357-359 i“ The integration of NTD case finding into the CHPS system represents a paradigm shift from population-level mass drug administration (MDA) toward individualized patient centered care “This sentence indicates as if this study gives guidance on MDA, I think based on the result of this study we cannot conclude anything about MDA

Response: We have revised the sections and removed the references to mass drug administration (MDA).
