# Supplementary material for: Integrating neglected tropical diseases into primary healthcare services in Ghana: Results from an initial pilot
Source: PLoS Negl Trop Dis. 2026 Oct 7;20(10):e0014792. doi: 10.1371/journal.pntd.0014792 (PMC13660979; doi:10.1371/journal.pntd.0014792)
Supplement: Supplementary file 3 [file pntd.0014792.s003.docx]

**11th August 2026**

**Kumasi Centre for Collaborative Research**

**Kwame Nkrumah University of Science and Technology**

**Kumasi, Ghana**

**The Editor**

**PLoS NTD Journal**

Dear Sir,

**Re: ‘Integrating Neglected Tropical Diseases into Primary Healthcare services in Ghana: results from an initial pilot’ PNTD-D-26-00911R1**

My co-authors and I have taken note of the review comments and revised the manuscript as suggested.

We wish to submit the revised manuscript for publication in your esteemed journal.

We look forward to the next steps towards the publication of our manuscript.

Yours Sincerely,

Yaw Ampem Amoako

Corresponding author

**Point-by-point response to reviewers’ comments**

Reviewer #1:

The objectives of the study are clearly articulated and the study design is appropriate for the stated objectives. The sample size is also acceptable and the statistical analysis is also good enough to support the conclusions for the pilot study. No formal hypothesis testing was peformed between districts.

Response: Thank you

Reviewer #2: The revised manuscript is substantially improved and the study design is broadly appropriate for its stated purpose as a descriptive implementation pilot assessing the feasibility of integrating syndromic NTD screening into routine CHPS activities. The objectives are now more clearly framed around feasibility, case identification, referral and referral completion, rather than hypothesis testing; accordingly, the absence of a formal sample-size calculation or inferential statistical testing is acceptable for this pilot design. The population and routine household-screening approach are also better described, and the revised analysis, including clearer denominators and confidence intervals for key referral proportions, is generally appropriate to support the descriptive conclusions.
I do not consider that additional major analyses are required. The remaining issues are mainly clarifications and moderation of interpretation: the authors should clearly distinguish household visits, individuals screened, indicator observations and referred individuals; ensure that percentage-based comparisons are not described as prevalence; further temper claims regarding epidemiological patterns, generalisability and readiness for national scale-up; and clarify the denominator used for assessing implementation among trained health workers. Ethical approval and written informed consent are reported, although consent procedures for minors and handling of identifiable referral data should be briefly clarified if applicable. Subject to these minor revisions, the methods and analyses are adequate for the study objectives.

Response: We are pleased that the reviewer finds the revised manuscript substantially improved and the study design, methods, and analyses appropriate for the objectives of this implementation pilot.

We included the distinction between household visits, indicator observations, and proportion of visits requiring referral and referral completion rates in the results section. We previously stated in the results section (line 332-334) that:

‘During the three-month pilot period, CHOs conducted a total of 1,932 home visits and successfully recorded 1,117 observations for seven pre-defined NTD-related clinical indicators.’

Additional details on the home visits and referral rates were also provided under the section on ‘Referral and Linkage to care’ (line 358-376) in the results section where we detailed the differential referral and referral completion rates across the study districts and in Table 3.

We have replaced the word ‘prevalence’ with ‘frequency’ to ensure that percentage-based comparisons are not presented as prevalence estimates (line 335, 339, 409), and tempered statements regarding epidemiological patterns, generalisability, and national scale-up (line 468-469).

We have revised figure 3 to reflect the correct numerator and denominator used for assessing implementation among trained Community Health Officers (CHOs). The numbers now reflect the proportion of CHOs who are implementing the training received.

We have also expanded the ethics section to clarify consent procedures for minors and the handling of identifiable referral data by adding the following sentences (line 157-166):

‘For participants younger than 18 years, written informed consent was obtained from a parent or legal guardian, with additional assent obtained from children 12 – 17 years. To facilitate referral and follow-up, limited personal identifiers, including participant name, age, sex, community of residence, contact information, and referral identification number, were collected and recorded on referral forms. For individuals less than 18 years old, the name and contact details of a parent or guardian were also recorded where necessary. These identifiable data were used solely for the purpose of clinical referral and care coordination and were accessible only to authorized study and health service personnel. All data were de-identified prior to analysis and reporting.’

Reviewer #3: By now the objective is clearly outlined. The study design is appropriate for the study objective. justification is provided for not calculating sample size. only thing which is missed is the justification for not including qualitative study. Probably this will be included in future studies

Response: We previously included sentences in the limitations section to address this concern as follows:

‘Also, while we documented the referral compliance gap, the study design did not include a qualitative component to explore the reasons behind patients' decisions to seek or not seek further care. This limits our ability to design targeted interventions to address this bottleneck.’ (line 490-493) and

‘Future research should employ implementation science frameworks to prospectively identify barriers and enablers of referral compliance from both the patient and health system perspectives.’ (line 505-507)

**Results**

Reviewer #1: The results were presented were clear and complete. The figures and tables were of sufficient quality.

Response: Thank you

Reviewer #2: The authors have corrected important numerical inconsistencies, clarified the denominator used in Table 2, consolidated referral outcomes in Table 3, and added counts and 95% confidence intervals for the main referral and referral-completion proportions. The tables and remaining figures are generally adequate for communicating the findings.
A few clarifications are still warranted before acceptance. Most importantly, the Results should consistently distinguish between home visits, individuals screened, indicator observations, individuals referred, and individuals completing referral, as these are not necessarily equivalent analytical units. Table 2 percentages should also be described as the distribution of recorded syndromic indicators across districts, rather than prevalence or epidemiological rates. Finally, the denominator underlying the health-worker implementation assessment should be clarified, since 87 workers were trained whereas the district-specific implementation figures refer to 71 workers.

Response: The title of Table 2 as been revised as suggested.

See also response to reviewer 2 above regarding query on home visits and referral completion rates.

Reviewer #3: By now the results are clearly and completely presented
The figures and tables have sufficient quality and clarity

Response: Thank you

**Conclusions**

Reviewer #1: The conclusions are supported by the data presented and are acceptable. The public health relevance of integrating NTDs into the PHC has also been incorporated.

Response: Thank you

Reviewer #2: The revised Conclusions and Discussion are substantially improved and are now largely aligned with the evidence generated by this descriptive implementation pilot. The authors appropriately emphasize the operational feasibility of incorporating syndromic NTD indicators into routine CHPS home visits, strong uptake by trained health workers, and the important gap between referral and completed facility attendance. The limitations section has also been strengthened to acknowledge the short implementation period, absence of population-level denominators, reliance on non-specific syndromic indicators without diagnostic confirmation, small numbers for some indicators, and the inability to investigate determinants of referral completion or longer-term sustainability.
The manuscript also discusses the contribution of these findings to understanding how an existing community health platform may support integrated NTD case detection and linkage to care, and the public health relevance is clearly articulated in relation to PHC, UHC and the NTD control and elimination agenda. Some conclusions should nevertheless be further moderated: statements suggesting readiness for wider or national implementation, strong transferability across Ghana, or broader regional generalisability remain stronger than can be supported by three purposively selected districts and a three-month pilot. These should be reframed as justification for broader prospective evaluation rather than demonstrated scalability.

Response: Thank you for the positive assessment of our manuscript.

We have also indicated in the discussion (line 468-469) that our findings require confirmation in larger studies in similar settings to ascertain scalability.

Reviewer #3: By now the comments have been addressed. The interpretation language has been revised throughout to ensure consistency with a descriptive analytical approach (as a pilot study)) and to avoid overinterpretation of the findings. However, I still believe there should be engagement of specialists like dermatologists to confirm the diagnosis made by CHOs

Response: We indicated in the limitations section (line 480-483) that the indicators used in the study were syndromic and that no diagnostic confirmation was available. This approach is consistent with real life practice at primary health care levels in Ghana where diagnostic testing and highly skilled experts like dermatologists are unavailable.

**Editorial and Data Presentation Modifications?**

Reviewer #1: None

Reviewer #2: I recommend Minor Revision. The manuscript has improved substantially and the remaining issues are primarily editorial or involve clarification of existing data rather than additional analyses.
The authors should ensure consistent terminology throughout, particularly distinguishing household visits, individuals screened, syndromic indicator observations, individuals referred, and referral completions. This distinction is important because one household visit may include several individuals and one individual may contribute more than one indicator. Relatedly, percentages in Table 2 should be described consistently as the distribution of recorded syndromic indicators across districts, rather than as prevalence or disease-specific rates.

Response: See response above to query by reviewer 2 regarding the results. The title of Table 2 has been modified as suggested and we have replaced the word ‘prevalence’ with ‘frequency’ or ‘proportion’ within the manuscript text.
The denominator used for the health-worker implementation assessment should also be clarified. Although 87 workers were trained overall, the district-specific implementation figures sum to 71 assessed workers. The manuscript should explain who was included in this assessment and why the denominator differs from the total trained population.

Response: We have revised figure 3 and the related text within the manuscript to indicate that figures referred to the proportion of Community Health Officers who implemented after completing the training.
A final editorial revision should moderate residual language implying epidemiological patterns, national scalability, or broad generalisability, and ensure that suggested interventions to improve referral completion are presented as potential strategies requiring evaluation rather than demonstrated solutions. The remaining figure/table presentation is generally adequate, although Table 2 and Table 3 captions and footnotes should make their denominators and analytical units explicit. Finally, a careful language and reference-formatting check is warranted to correct remaining grammatical inconsistencies and incomplete reference entries. Subject to these relatively minor revisions, the manuscript would be suitable for acceptance.

Response: We have deleted the word ‘epidemiological’ from the discussion section as suggested.

We have also indicated in the discussion (line 468-469) that our findings require confirmation in larger studies in similar settings to ascertain scalability.

Further, the title of Table 2 has been modified as suggested.

Reviewer #3: In view of the authors' thorough and diligent responses to the reviewers' comments and their explicit acknowledgment of the study's limitations with respect to the generalizability of the findings, I have decided to accept this manuscript for publication

Response: Thank you

**Summary and General Comments**

Reviewer #1: Acceptable for publication.

Response: Thank you

Reviewer #2: The manuscript addresses an important implementation question on integrating syndromic NTD screening and referral into Ghana’s CHPS platform. Its main strengths are the pragmatic use of an existing primary healthcare system, stakeholder engagement, strong CHO uptake, and the identification of a substantial referral-completion gap. The revised manuscript is considerably improved, particularly in the description of screening procedures, referral ascertainment, and study limitations.
The remaining issues are mainly minor. The authors should clearly distinguish household visits, individuals screened, indicator observations, referrals and completed referrals; avoid describing syndromic indicator distributions as prevalence or epidemiological patterns; clarify the denominator used for the health-worker implementation assessment; and further moderate claims regarding scalability and generalisability.
Overall, the study provides useful operational evidence with clear public health relevance. I recommend Minor Revision.

Response: Thank you. We have revised the manuscript to address the review concerns as detailed above

Reviewer #3: We would like to extend our sincere gratitude to the authors for their thorough dedication in addressing nearly all of the reviewers’ comments. We believe this pilot study will serve as a critical foundation for a future mixed-methods implementation study, ultimately generating the necessary evidence to support the integration of NTD care into primary healthcare (PHC) systems.

Response: Thank you
