# Supplementary material for: Integrating neglected tropical diseases into primary healthcare services in Ghana: Results from an initial pilot
Source: PLoS Negl Trop Dis. 2026 Oct 7;20(10):e0014792. doi: 10.1371/journal.pntd.0014792 (PMC13660979; doi:10.1371/journal.pntd.0014792)
Supplement: Supplementary file 4 [file pntd.0014792.s004.docx]

**6th September 2026**

**Kumasi Centre for Collaborative Research**

**Kwame Nkrumah University of Science and Technology**

**Kumasi, Ghana**

**The Editor**

**PLoS NTD Journal**

Dear Sir,

**Re: ‘Integrating Neglected Tropical Diseases into Primary Healthcare services in Ghana: results from an initial pilot’ PNTD-D-26-00911R2**

My co-authors and I have taken note of the review comments and revised the manuscript as suggested.

We wish to submit the revised manuscript for publication in your esteemed journal.

We look forward to the next steps towards the publication of our manuscript.

Yours Sincerely,

Yaw Ampem Amoako

Corresponding author

**Point-by-point response to reviewers’ comments**

Reviewer #2:

The Methods are generally appropriate for the study’s stated purpose as a descriptive implementation pilot, and the objectives are sufficiently clear within that feasibility framework. A formal testable hypothesis, sample-size calculation, or inferential comparison is not essential for this type of pilot, provided the conclusions remain descriptive and proportionate to the design. The population and routine CHPS screening procedures are adequately described, and the statistical approach is broadly appropriate.

The main issue still requiring clarification is the unit of analysis and denominator structure. The manuscript should clearly distinguish household visits, individuals screened, indicator observations, individuals referred and referral completions, and ensure that referral proportions are calculated using compatible numerator and denominator units. The denominator for the health-worker implementation assessment should also be reconciled. No major ethical concerns are apparent, although consent procedures for minors and handling of identifiable referral data should be clarified if applicable.
Overall, no major new analyses are required; the remaining issues are methodological clarifications that should be resolved before acceptance.

Response: We thank the reviewer for the overall positive review comments. The ethical considerations regarding consent procedures for minors (12-17 years) and handling of identifiable referral data were previously included in the ethics section (line 157-166)

**Results**
Reviewer #2: The Results are broadly consistent with the stated descriptive analysis plan, and the revised tables are clearer than in the original submission. However, some numerical presentation issues remain and should be resolved before acceptance. In particular, the manuscript still mixes household visits, indicator observations, referred individuals and referral completions as analytical units, which makes some derived proportions difficult to interpret.

The main figures requiring clarification are:

- 1,932 home visits
- 1,117 indicator observations
- 615 referrals
- 415 completed referrals overall, derived from 4 + 130 + 281
- District referral proportions reported as 62/672 = 9.2%, 248/409 = 60.6%, and 305/851 = 35.8%

The key concern is that these referral proportions use home visits as the denominator, while the numerator is described elsewhere as individuals referred. Since several household members could be screened during one visit and one individual could contribute more than one indicator, numerator and denominator may represent different units. This needs to be clarified or recalculated using a compatible denominator.

Response: We have revised the manuscript to explicitly state that the reported proportions represent the number of referrals generated relative to the number of household visits conducted.

We have included the following sentence in the results section (line 361-365):

‘Referral proportions were calculated as the number of referrals generated per household visit conducted in each district. Because multiple individuals may be screened during a single household visit, these proportions reflect referrals relative to household visits rather than the proportion of individuals referred.’

A second issue is Table 2. Its footnote correctly states that percentages represent the proportion of all recorded observations for each indicator contributed by each district and are not prevalence estimates. However, the text still describes figures such as 59.3% skin lesions in Ga South, 81.6% visible deformities in Ga South, and 75% blood in urine in Ga South using terms such as “prevalence” or “most prevalent.” These are distributional proportions, not district prevalence estimates, and the wording should be corrected accordingly.

Response: We have revised the text throughout the manuscript and removed terms such as “prevalence,” “most prevalent,” and similar expressions when referring to these percentages. The Results section now describes these findings as the distribution of recorded indicator observations across districts. The sections now read:

‘*Skin lesions* were the most common NTD-related clinical indicator reported across all three study sites. Ga South accounted for 59.3% (386/651) of all recorded skin lesion observations, followed by Nkwanta South with approximately 32.9% (Table 2).

Ga South accounted for 81.6% (62/76) of *visible deformities* followed by Kassena Nankana West (18.4%), with no cases documented in Nkwanta South (Table 2). The district located in Northern Ghana, Kassena Nankana West, accounted for 81 of 120 (67.5%) recorded eye-related complications and 135 of 167 (80.8%) recorded lymphoedema observations.’ (line 336-344)

‘Notably, the coastal Ga South district accounted for 75% (15/20) of the total recorded observations of the presence of *blood in urine*, a key indicator which may suggest urogenital schistosomiasis.’ (line 348-350)

Finally, the health-worker implementation results remain internally unclear: 87 workers were trained, whereas the district-specific implementation denominators are 21 + 28 + 22 = 71, with 63 reported as implementing. The manuscript should explain why 16 trained workers are not represented in this assessment and define exactly which cadres are included.

Response: To address the review concern, we have revised the results section to now read (line 385-391):

‘A total of 87 healthcare workers (including CHOs and supervisory staff) were trained across the three districts as part of the pilot preparations; however, the implementation assessment was conducted only among cadres (CHOs, public health nurses and disease control officers) directly responsible for carrying out community screening and referral activities during the period. The trained workers who were not directly involved in implementation activities and/or were not available for follow-up assessment during the evaluation period were not included in this analysis.’

Overall, the tables and figures are of acceptable quality, but these inconsistencies in analytical units and denominators should be addressed before publication.

Response: Thank you. The analytical units and denominators have been revised as detailed above.

**Conclusions**
Reviewer #2: The Conclusions are generally supported by the data insofar as they emphasize the operational feasibility of incorporating syndromic NTD indicators into CHPS activities, strong uptake by CHOs, and the substantial referral-completion gap. The limitations are now clearly described, including the lack of population-level denominators, absence of diagnostic confirmation, possible misclassification, small numbers for some indicators, and the short duration of the pilot. The manuscript also explains the public health relevance of strengthening community-based NTD detection and linkage to care within PHC and UHC.

However, some statements still exceed the evidence. Claims concerning “epidemiological patterns,” readiness for wider or national implementation, enhanced transferability across Ghana, and broader generalisability to sub-Saharan Africa should be further moderated. Given the purposive selection of only three districts, absence of representative population denominators, and lack of diagnostic validation, the findings are better framed as proof-of-concept operational evidence that justifies broader evaluation, rather than evidence of established scalability or generalisability.

Overall, the conclusions are close to acceptable, but these overinterpretations should be corrected before publication.

Response: We have deleted terms such as “epidemiological patterns,” “readiness for wider implementation,” “transferability,” and “generalisability” as suggested. We have further revised the section to now read (line 518-534):

‘**Conclusion**

This pilot demonstrates that integrating Neglected Tropical Diseases (NTDs) into Ghana’s primary healthcare system through the CHPS platform is feasible and operationally practical. Incorporating simple NTD indicators into routine home visit registers enabled community health officers to detect suspected cases and generate useful, location-specific data across diverse settings. Our findings provide proof-of-concept operational evidence demonstrating the feasibility and acceptability of incorporating syndromic NTD indicators into routine CHPS activities, the strong engagement of Community Health Officers, and the importance of addressing gaps in referral completion. However, large disparities in referral rates and referral compliance underscores persistent health system and community-level barriers to effective linkage to care that could limit health impact. Addressing community- and system-level barriers to referral completion will be essential. Overall, integrating NTD services into PHC offers a promising pathway toward Universal Health Coverage and achievement of Ghana’s NTD control and elimination goals, provided that detection is matched by timely and effective care. Our findings support the need for larger studies to evaluate effectiveness, implementation requirements, and scalability in diverse settings.’

**Editorial and Data Presentation Modifications?**
Reviewer #2: I recommend Minor Revision. The manuscript is substantially improved, and the remaining issues are primarily editorial and related to clarification of existing data rather than requiring new fieldwork or major analyses.

The authors should ensure consistent use of analytical units throughout the Results, clearly distinguishing home visits, individuals screened, indicator observations, individuals referred, and referral completions. In particular, the referral proportions in Table 3 should be checked to ensure that numerator and denominator refer to compatible units. The health-worker implementation denominator should also be clarified, since 87 workers were trained but the district-specific implementation assessment includes only 71.

Table 2 is now much clearer, but the accompanying text should avoid terms such as “prevalence” or “epidemiological patterns”, because the reported percentages represent the distribution of syndromic indicator observations across districts rather than population-based prevalence estimates. Similarly, statements regarding national scale-up, transferability across Ghana, or generalisability to other countries should be further moderated and framed as areas for future evaluation.

Response: We thank the reviewer for the positive assessment of the manuscript. We have revised the manuscript to ensure consistent use of analytical units throughout the Results section, clearly distinguishing between home visits, indicator observations, referred individuals, and completed referrals. In addition, we have clarified that although 87 health workers were trained, the implementation assessment was conducted among the subset of 71 eligible and available workers, and we now explicitly define the cadres included.

We further revised the text accompanying Table 2 to avoid terms such as “prevalence,” “most prevalent,” and “epidemiological patterns,” which could imply population-based estimates. These findings are now consistently described as the distribution of syndromic indicator observations across districts.

We have moderated statements regarding scalability, transferability, and generalisability, framing the findings as proof-of-concept operational evidence from three purposively selected districts that supports the need for broader evaluation in more diverse settings.

We believe these revisions improve the clarity, accuracy, and interpretative balance of the manuscript and fully address the reviewer's concerns.

A final editorial review is recommended to address several residual language and formatting issues. Examples include subject–verb agreement (“versions… was piloted”; “disparities… underscores”), grammatical errors (“could possibility result”), inconsistent terminology (“primary healthcare/primary health care”; “sub-national/subnational”), and continued use of “prevalence” for percentages that Table 2 correctly defines as distributions of syndromic observations. Reference 23 is also incomplete (“2021 Census reports:[”), and the formatting of organizational/online references should be standardized. These are minor editorial issues but should be corrected before publication.

Response: We conducted a comprehensive editorial review of the manuscript as suggested to correct grammatical errors.

In addition, we carefully reviewed the Results and Discussion sections to ensure that terms such as “prevalence” are no longer used when referring to the percentages presented in Table 2. These percentages are now consistently described as the distribution of recorded syndromic indicator observations across districts, which more accurately reflects the nature of the data and the analysis conducted.

We have also corrected and completed Reference 23 as suggested.

**Summary and General Comments**
Reviewer #2: The manuscript addresses a relevant implementation question and provides useful operational evidence on incorporating syndromic NTD screening into Ghana’s CHPS platform. Its main strengths are the pragmatic use of an existing primary healthcare structure, stakeholder engagement, CHO participation, and the identification of an important referral-completion gap. The revised manuscript is substantially improved compared with the original submission, particularly in the description of screening procedures, referral ascertainment, presentation of referral outcomes, and acknowledgement of key limitations.

The remaining weaknesses are mainly related to clarity and interpretation rather than study execution. The authors should resolve the unit-of-analysis issue across home visits, individuals screened, indicator observations and referrals; clarify the denominator for the health-worker implementation assessment; avoid describing syndromic indicator distributions as prevalence or epidemiological patterns; and further temper claims regarding scalability and generalisability. No new experiments or major analyses are required.

Overall, the study is programmatically relevant and potentially publishable after these final clarifications. I recommend Minor Revision.

Response: Thank you. The manuscript has been revised as suggested.
