# Supplementary material for: Integrating neglected tropical diseases into primary healthcare services in Ghana: Results from an initial pilot
Source: PLoS Negl Trop Dis. 2026 Oct 7;20(10):e0014792. doi: 10.1371/journal.pntd.0014792 (PMC13660979; doi:10.1371/journal.pntd.0014792)
Supplement: Supplementary file 5 [file pntd.0014792.s005.docx]

28th September 2026

**Kumasi Centre for Collaborative Research**

**Kwame Nkrumah University of Science and Technology**

**Kumasi, Ghana**

The Editor,

PLOS NTD Journal

Dear Sir,

**Re: ‘Integrating Neglected Tropical Diseases into Primary Healthcare services in Ghana: results from an initial pilot’ PNTD-D-26-00911R3**

My co-authors and I are grateful to the Editors and reviewer for making time to review our manuscript. We have read the reviewer comments and wish to respond as detailed below.

We look forward to the next steps towards the publication of our manuscript.

Yours Sincerely,

**Yaw Ampem Amoako**

**Corresponding author**

**Point-by-point response to review comments**

Reviewer #2: The Methods are now appropriate for the study’s stated purpose as a descriptive implementation pilot. The objectives, study population, routine CHPS screening procedures, descriptive analytical approach, and ethical safeguards are sufficiently described, and no additional major analyses or sample-size calculations are required for acceptance. The lack of formal hypothesis testing is acceptable given the feasibility-focused design.
The previous concerns regarding consent for minors and handling of identifiable referral data have also been addressed. The only remaining methodological issue is largely terminological: the referral measure should be described consistently as referrals generated relative to household visits, rather than implying an individual-level referral proportion. This is now explained in the Results and does not require further analysis

Response: Thank you.

**Results**

Reviewer #2: The Results are now consistent with the descriptive analysis plan and are presented clearly overall. The revised text distinguishes indicator observations from prevalence estimates, explains the referral metric more transparently, and presents referral completion with appropriate counts and confidence intervals. The tables and figures are generally of sufficient quality to support interpretation.
Only minor wording could still be improved, particularly by describing the referral measure consistently as referrals generated relative to household visits rather than as an individual-level referral proportion. This does not require additional analysis and would not prevent acceptance. See comments provided to authors.

Response: The section has been revised as suggested (lines 365 and line 378).

**Conclusions**

Reviewer #2: The Conclusions are now well aligned with the data presented. The authors appropriately emphasize the feasibility of incorporating syndromic NTD indicators into routine CHPS activities, the strong engagement of CHOs, and the importance of addressing referral-completion gaps. The limitations are clearly acknowledged, including the short duration, absence of population-level denominators, lack of diagnostic confirmation, possible misclassification, and limited ability to assess determinants of referral completion or long-term sustainability.
The manuscript also explains how these findings contribute to the operational evidence base for integrating NTD detection and referral into primary health care, and the public health relevance is clearly articulated in relation to UHC and NTD control and elimination. The revised Conclusion is appropriately cautious and frames the findings as proof-of-concept evidence requiring evaluation in larger studies. I do not consider further substantive revision necessary.

Response: Thank you.

**Editorial and Data Presentation Modifications?**
Reviewer #2: I recommend Accept with minor editorial corrections. The manuscript has improved substantially across successive revisions, and the remaining issues do not affect the validity of the findings or require additional analyses.
A few minor editorial refinements would further improve clarity: consistently describe the referral metric as referrals generated relative to household visits; clarify that the implementation percentages presented in Figure 3 refer to CHOs; remove or soften the remaining “mixed-methods” wording unless qualitative methods/results are formally presented; and ensure that residual statements about transferability, generalisability, and national scale-up remain aligned with the cautious framing adopted in the revised

Response:

We have revised the results section to ensure that the referral metric described the referrals generated relative to household visits. (lines 365 and line 378).

We have also clarified that the percentages presented in figure 3 refers to community health officers (CHOs) (line 398-399); this was also already reflected in the legend of figure 3.

We reviewed the manuscript for remaining references to a “mixed-methods” design and moderated this terminology (line 483-486).

We revised statements regarding transferability, generalisability, and potential national scale-up to ensure they remain consistent with the interpretation adopted in the revised manuscript, emphasizing the exploratory nature of the findings and the need for further evaluation in broader settings. The section now reads (line 473-475):

‘While the pilot focused on NTDs with visible signs, the findings suggest that task-sharing surveillance to CHOs could have broader applicability; however, further research is needed to determine its effectiveness and feasibility in other contexts.’

**Summary and General Comments**
Reviewer #2: I identified only a few residual inconsistencies, which I would treat as editorial:
- Referral terminology: because several people could be screened during one household visit, “referrals per household visit” or “referrals per 100 household visits” would be technically clearer than “proportion of home visits leading to referrals” unless the authors can confirm a maximum of one referral per visit.

Response: The section has been revised as suggested (lines 365 and line 378).

- Implementation denominator: the revised paragraph says the assessment included CHOs, public health nurses and disease control officers, but the district denominators reported (21, 25 and 18) correspond to the 64 CHOs, and Figure 3 is explicitly labelled as CHOs. The text should simply state that the implementation percentages presented refer to CHOs.

Response: We have revised the results section to ensure that the referral metric described the referrals generated relative to household visits. (lines 365 and line 378).

We have also clarified that the percentages presented in figure 3 refers to community health officers (CHOs) (line 398-399); this was already reflected in the legend of figure 3.

- Some earlier wording remains stronger than the revised Conclusion. The Discussion still says the three districts “enhance the transferability” of the findings, that task-sharing is “highly generalizable,” and refers to national scale-up/phased national roll-out. Given that the Conclusion now appropriately calls for larger studies to evaluate scalability, these residual phrases could be softened for consistency.

Response: The sentences have been revised appropriately and now read:

‘The inclusion of districts from Ghana's northern, middle, and southern zones enhances the diversity of contexts represented in the study, providing preliminary evidence on the intervention's applicability across varied ecological and socio-cultural settings.’ (line 462-465)

‘While the pilot focused on NTDs with visible signs, the findings suggest that task-sharing surveillance to CHOs could have broader applicability; however, further research is needed to determine its effectiveness and feasibility in other contexts.’ (line 472-475):

- The Discussion still calls the monitoring a “mixed-methods monitoring approach”, although the limitations state that the study did not include a qualitative component. I would simply remove “mixed-methods” or describe this as monitoring incorporating qualitative feedback.

Response: Done as suggested. The section now reads (line 483-486):

‘The monitoring approach assessed both quantitative outputs (signs, referrals) and incorporated qualitative feedback on tool usability, thereby providing a comprehensive understanding of the implementation process.’
