# Supplementary material for: Integrating neglected tropical diseases into primary healthcare services in Ghana: Results from an initial pilot
Source: PLoS Negl Trop Dis. 2026 Oct 7;20(10):e0014792. doi: 10.1371/journal.pntd.0014792 (PMC13660979; doi:10.1371/journal.pntd.0014792)
Supplement: Peer review history

# Peer review: Decision Letter 0

**DOI:** 10.1371/journal.pntd.0014792.r001

**Authors:** Stuart D. Blacksell, Kebede Deribe

29 Jun 2026

PNTD-D-26-00911

Mainstreaming Neglected Tropical Diseases into Primary Healthcare services in Ghana: results from an initial pilot

PLOS Neglected Tropical Diseases

Dear Dr. Amoako,

Thank you for submitting your manuscript to PLOS Neglected Tropical Diseases. After careful consideration, we feel that it has merit but does not fully meet PLOS Neglected Tropical Diseases's publication criteria as it currently stands. Therefore, we invite you to submit a revised version of the manuscript that addresses the points raised during the review process.

Please submit your revised manuscript within by Aug 28 2026 11:59PM. If you will need more time than this to complete your revisions, please reply to this message or contact the journal office at plosntds@plos.org. When you're ready to submit your revision, log on to https://www.editorialmanager.com/pntd/ and select the 'Submissions Needing Revision' folder to locate your manuscript file.

Please include the following items when submitting your revised manuscript:

* A letter that responds to each point raised by the editor and reviewer(s). You should upload this letter as a separate file labeled 'Response to Reviewers'. This file does not need to include responses to any formatting updates and technical items listed in the 'Journal Requirements' section below.

* A marked-up copy of your manuscript that highlights changes made to the original version. You should upload this as a separate file labeled 'Revised Manuscript with Track Changes'.

* An unmarked version of your revised paper without tracked changes. You should upload this as a separate file labeled 'Manuscript'.

If you would like to make changes to your financial disclosure, competing interests statement, or data availability statement, please make these updates within the submission form at the time of resubmission. Guidelines for resubmitting your figure files are available below the reviewer comments at the end of this letter.

As the corresponding author, your ORCID iD is verified in the submission system and will appear in the published article. PLOS supports the use of ORCID, and we encourage all coauthors to register for an ORCID iD and use it as well. Please encourage your coauthors to verify their ORCID iD within the submission system before final acceptance, as unverified ORCID iDs will not appear in the published article. Only the individual author can complete the verification step; PLOS staff cannot verify ORCID iDs on behalf of authors.

We look forward to receiving your revised manuscript.

Kind regards,

Kebede Deribe, BSc, MPH, PhD

Guest Editor

PLOS Neglected Tropical Diseases

Stuart Blacksell

Section Editor

PLOS Neglected Tropical Diseases

Shaden Kamhawi

co-Editor-in-Chief

PLOS Neglected Tropical Diseases

orcid.org/0000-0003-4304-636XX

Paul Brindley

co-Editor-in-Chief

PLOS Neglected Tropical Diseases

orcid.org/0000-0003-1765-0002

**Additional Editor Comments (if provided):**

**Journal Requirements:**

If the reviewer comments include a recommendation to cite specific previously published works, please review and evaluate these publications to determine whether they are relevant and should be cited. There is no requirement to cite these works unless the editor has indicated otherwise.

1) Thank you for including an Ethics Statement for your study. Please include:

i) A statement that formal consent was obtained (must state whether verbal/written) OR the reason consent was not obtained (e.g. anonymity). NOTE: If child participants, the statement must declare that formal consent was obtained from the parent/guardian.].

2) Please amend your detailed Financial Disclosure statement. This is published with the article. It must therefore be completed in full sentences and contain the exact wording you wish to be published.

i) State the initials, alongside each funding source, of each author to receive each grant. For example: "This work was supported by the National Institutes of Health (####### to AM; ###### to CJ) and the National Science Foundation (###### to AM).".

If you did not receive any funding for this study, please simply state: u201cThe authors received no specific funding for this work.u201d

**Reviewers' Comments:**

Reviewer's Responses to Questions

**Key Review Criteria Required for Acceptance?**

As you describe the new analyses required for acceptance, please consider the following:

**Methods**

-Are the objectives of the study clearly articulated with a clear testable hypothesis stated?

-Is the study design appropriate to address the stated objectives?

-Is the population clearly described and appropriate for the hypothesis being tested?

-Is the sample size sufficient to ensure adequate power to address the hypothesis being tested?

-Were correct statistical analysis used to support conclusions?

-Are there concerns about ethical or regulatory requirements being met?

Reviewer #1: The objectives of the study are described in terms of detecting clinical signs of some selected NTDs by means of integrated surveillance system with trained community health officers (CHOs) for subsequent referral and linkage to the nearby health facilities. However, this characterization doesn't imply proper articulation of testable hypothesis. It's not clear as well whether the study design is meant to test the effectiveness of "integrated NTD surveillance" or "mainstreaming of NTDs into the PHC system". Also, it is not clear whether the study settings and health facilities selected were known endemic districts for the 7 target NTDs. Trachoma is eliminated as a public health problem in Ghana several years ago and it's one of the 7 infections/conditions selected for integrated surveillance. There was no explanation about the sample size and the plan for statistical analysis.

Reviewer #2: The study is framed as an implementation pilot, and its design is broadly appropriate to that purpose: a descriptive, multi-zone deployment embedded in a routine community platform is a reasonable way to test whether CHOs can operationalise integrated NTD syndromic screening. However, the objectives are stated programmatically rather than as a testable hypothesis, with no predefined feasibility criteria or success thresholds (lines 35–38, 140–143), so the findings are narrated post hoc rather than judged against an a priori benchmark. The design is also uncontrolled, with no baseline or non-integrated comparator, and the qualitative monitoring component referred to in the Methods is not actually reported—a notable gap given that the referral-compliance disparity is the headline finding.

The population is described at district level (lines 161–175) but not at the analytic unit level: the within-subdistrict sampling frame is unspecified and, critically, no denominator is defined for the screened population, so the 1,932 visits and 1,117 observations cannot be situated against an eligible base and representativeness cannot be assessed. Sample size and power are not formally addressed—defensible for a feasibility pilot, but several key indicators rest on very small counts (blood in urine n=20; hydrocele n=42) yet are reported with confident percentages and comparative language. The statistical approach (descriptive analysis only) is appropriate in type but is not matched by appropriate caution: proportions are presented without confidence intervals, Table 2 denominators are ambiguous, and comparative claims imply contrasts that were never formally tested. The study is adequately sized to demonstrate feasibility and CHO uptake, but underpowered to support the district-level epidemiological contrasts and zone-attributable explanations advanced in the Discussion.

On ethical and regulatory requirements, the essentials are in place—institutional ethics approval, local permissions, and informed consent are reported (lines 145–151)—but the approval number (CHRPE/AP/335/19) appears to predate the 2023 implementation by several years and should be confirmed as covering this activity. Given household screening for sensitive signs, the authors should also clarify consent procedures for minors and how identifiable referral data were handled. In sum, the methods are fit for the narrow feasibility purpose but are described with insufficient precision and interpreted more strongly than the descriptive, uncontrolled, purposively sampled design can support.

Reviewer #3: -The objectives of the study clearly articulated with a clear testable hypothesis stated but do not align with the study title

-In the study design it would have been better if qualitative component included to determine enablers and barriers

-The population clearly described and appropriate for the hypothesis being tested

Repetitiveness is lacking, the western and Eastern part of the country has not been represented and also proper justification has not been provided

IRB clearance and informed consent procedures has been met.

**********

**Results**

-Does the analysis presented match the analysis plan?

-Are the results clearly and completely presented?

-Are the figures (Tables, Images) of sufficient quality for clarity?

Reviewer #1: The analysis lacks depth and clarity. The authors presented the results of the integrated surveillance with the proxy indicators they outlined for the CHOs. Not clear whether the 7 target NTDs were clearly described for the CHOs. The results were discussed as clinical signs presented by visible indications (observation) without additional investigations by means of interviews or other simple diagnostic means (using recognition cards, etc). The figures and tables used are too shallow and do not represent substantive evidence and statistical data.

Reviewer #2: The results are broadly consistent with the stated descriptive analysis plan—frequencies of the seven indicators by district, referral proportions, and implementation rates—so there is no evidence of analyses beyond what the Methods described. The limitation is the reverse: the descriptive plan is executed with comparative and quasi-inferential language ("most prevalent," "marked geographical variation," lines 293–302) that implies contrasts never formally tested. The analysis as planned is appropriate; the interpretation layered onto it is not fully matched by the plan.

The results are not yet clearly or completely presented, and this is the most consequential weakness. Percentages are reported against ambiguous denominators—Table 2 cell values cannot be reconciled to a single base (column total, district row total, or screened population), so the same figure carries different meanings depending on the reader's assumption (e.g., Nkwanta South skin lesions are shown as 23.4% against n=651 but represent 214/248 of that district's recorded indicators). Several in-text values do not match the tables or figures, and the visit and referral denominators (672, 409, 851; lines 316–318) do not align cleanly with the facility structure described earlier or with the 1,932 total. Key quantities also rest on very small counts reported without uncertainty (blood in urine n=20; hydrocele n=42), and a single derived metric central to the referral pathway—the positive predictive value of CHO identification—is referred to but never presented. Completeness requires counts alongside every percentage, a single labelled denominator per table column, exact text–table–figure correspondence, and 95% confidence intervals for the headline proportions.

The figures and tables are adequate in concept but not in execution. Table 1 is essentially a facility inventory that could be condensed or moved to a supplement. Table 2 needs explicit denominators in each column header to be interpretable. Figures 3–5 lack value labels and confidence intervals, and their axis counts only approximate the in-text numbers; Figure 4's legend ("Facility visits from referrals" vs "Home Referrals to health facility") is ambiguous as to which bar is numerator and which is denominator, and the trained-worker denominators differ between Methods (64 CHOs) and Results (87 workers), leaving Figure 5's base unclear. None of these are quality problems that require new data—they are matters of labelling, reconciliation, and the addition of denominators and uncertainty—but they must be resolved before the descriptive findings can be independently verified.

Reviewer #3: the results are clearly presented and the tables/figures have suffient quality

**********

**Conclusions**

-Are the conclusions supported by the data presented?

-Are the limitations of analysis clearly described?

-Do the authors discuss how these data can be helpful to advance our understanding of the topic under study?

-Is public health relevance addressed?

Reviewer #1: The conclusions are as good as the results discussed above. Nothing substantive as it pertains to mainstreaming of NTDs but something about integrated surveillance and referral linkages.

Reviewer #2: The conclusions are only partially supported by the data presented. The core claim—that integrating NTD syndromic indicators into the CHPS home-visit register is operationally feasible and is taken up by CHOs (lines 422–428)—is reasonably supported by the high implementation rate and the volume of indicator capture. The identification of a referral-completion bottleneck is likewise a fair reading of the compliance disparity. However, several conclusions extend beyond the evidence: the characterisation of the approach as "a paradigm shift from population-level MDA toward individualized patient-centered care" (lines 357–359), the readiness for "phased national roll-out" (lines 405–406), and the disease-specific epidemiological patterns inferred from non-specific proxy signs all outpace what a short, uncontrolled, purposively sampled descriptive pilot can demonstrate. No data on case confirmation, treatment, morbidity outcomes, or cost were generated, so the implied health and efficiency benefits are hypothesised rather than shown. The conclusions need to distinguish explicitly what was demonstrated (feasibility of integrated data capture, high CHO uptake, a referral gap) from what is hoped (scalability, health impact, the efficacy of proposed remedies).

The limitations are described, and the authors deserve credit for candour: they acknowledge the short three-month window, the reliance on suspected cases without confirmatory diagnostics, the absence of a qualitative component to explain referral behaviour, and the inability to assess long-term sustainability (lines 394–403). This is a genuine strength relative to many implementation reports. The limitations section is nonetheless incomplete in the areas that most affect validity—it does not address the undefined population denominator, the ambiguous percentage bases, the small cell counts underlying several headline figures, or the misclassification risk inherent in mapping single syndromic signs to specific diseases. Folding these into the limitations would materially strengthen the manuscript's internal consistency.

The authors do engage with how the data advance understanding, and they do so credibly within the feasibility frame: the work shows that an existing routine community platform can be repurposed for integrated NTD surveillance, that frontline uptake is achievable, and that detection alone is insufficient without active linkage to care. This is a useful contribution to the operational evidence base, and the candid surfacing of the referral-compliance gap is its most transferable insight. The advance would be stronger still if the discussion separated demonstrated feasibility from the broader inferential and policy claims, and framed the district-level explanations as hypotheses for prospective testing.

Public health relevance is clearly and appropriately addressed. The manuscript situates the work within the WHO 2030 NTD roadmap, the UHC agenda, and Ghana's NTD master plan (lines 108–123, 341–353), and it draws actionable programmatic implications—patient navigation, transport subsidies, audit-and-feedback learning from the high-performing district (lines 405–413). These are sensible and relevant, provided they are presented as plausible, untested options rather than validated solutions. On balance, the conclusions are relevant and partly supported, but require moderation to align with the descriptive design, and the limitations require modest expansion to cover the denominator, classification, and uncertainty issues.

Reviewer #3: the conclusion supports the data presented but in some it doesn't . Example The recommendation about MDA is not supported by study data and do not align with the study objectives

The public health relevance of the study has been clearly stated

**********

**Editorial and Data Presentation Modifications?**

Use this section for editorial suggestions as well as relatively minor modifications of existing data that would enhance clarity. If the only modifications needed are minor and/or editorial, you may wish to recommend “Minor Revision” or “Accept”.

Reviewer #1: Major revisions required.

Reviewer #2: Minor Comments

Abstract

• The Abstract reports “67% compliance” overall (line 52) but the Results give district-specific compliance ranging from <10% to 92.1% (lines 322–325); an aggregate figure masking this range is misleading and should be removed or contextualised.

• State the study design explicitly as a three-district descriptive implementation pilot, and avoid the disease-specific attributions (“suggestive of schistosomiasis”, line 54) in favour of syndromic wording.

• Clarify whether “1,117 suspected cases” (line 51) refers to individuals or to indicator observations, since an individual may present more than one sign.

Introduction

• The count of endemic NTDs is stated as 21 globally (line 90) and 14 in Ghana (line 98); confirm against the current WHO list (now 21 conditions/disease groups) and ensure consistency with the GHS NTD Master Plan reference [9].

• The claim that NTDs are “entirely preventable” (line 96) is an overstatement for several conditions; soften to “preventable and controllable.”

• Reference [9] is dated 2021–2025; verify it remains the current master plan or note the successor document.

Methods

• Specify the sampling frame within each subdistrict (how households and individuals were selected for the home visit) and whether all household members were screened (lines 155–169).

• The operational definition of hydrocele as “any scrotal swelling” (line 182) and lymphoedema as “swollen limbs” (line 187) are very broad; note expected specificity limitations.

• State how referral compliance was ascertained (facility register linkage, CHO follow-up, or patient self-report) and the follow-up window (lines 312–325).

• Indicate whether the electronic and paper-based registers produced concordant data, given both were piloted (lines 137–138).

• Software is listed as GraphPad Prism 6.0 and Stata 12 (lines 272–274); for a descriptive analysis, clarify what each tool was used for, and note that these are dated versions.

• The ethics approval number (CHRPE/AP/335/19; line 149) predates the 2023 implementation by several years—confirm the approval covers this activity or cite an amendment.

Results

• Add the denominator to every Table 2 column header and reconcile the percentage base (column vs row); the current presentation is ambiguous (lines 309–310).

• Report counts with all percentages in the text (e.g., eye complications “~70%” and lymphoedema “81%” in KNW; lines 300–302).

• Figures 3–5 lack value labels and confidence intervals; axis counts should match in-text numbers exactly.

• Fig 4 legend labels (“Facility visits from referrals” vs “Home Referrals to health facility”) are confusing—clarify which bar is numerator and which is denominator.

• The PPV of CHO identification is not reported despite confirmation being central to the referral pathway; if facility confirmation data exist, present them.

• The training/implementation denominators differ between the Methods (64 CHOs trained; line 220) and Results (87 workers trained; line 329)—reconcile and define which group Fig 5 describes.

Discussion

• Avoid “validating the ability of CHOs to act as effective surveillance sentinels” (lines 350–351); the pilot did not validate accuracy against a reference standard.

• The reverse-causality and confounding caveats applied to district differences should be stated explicitly rather than implied.

• The limitations paragraph (lines 394–403) is appropriate but should be expanded to include the denominator and misclassification issues raised in the major comments.

• Temper “paradigm shift” (line 357) and “dismally low” (line 365) toward neutral scientific register.

Language and Presentation

• Standardise terminology: “lymphoedema” vs “lymphedema,” “sub-national” vs “subnational,” and “Kassena Nankana” vs “Kassena-Nanka” (Table 1 header, line 288).

• Several sentences are incomplete or have agreement errors (e.g., “The recorded NTD related data as captured…”, lines 228–229); a careful proofread is needed.

• Define all abbreviations at first use (ICCC, CHIM, MMDP) and ensure consistent expansion.

• Ensure figure callouts (Fig 1–Fig 5) appear in numerical order and that all figures are cited in the text.

• Check reference formatting and access dates (several references list future-dated access notes, e.g., “12th March, 2026”).

Reviewer #3: Has been stated above, it requires revision

**********

**Summary and General Comments**

Use this section to provide overall comments, discuss strengths/weaknesses of the study, novelty, significance, general execution and scholarship. You may also include additional comments for the author, including concerns about dual publication, research ethics, or publication ethics. If requesting major revision, please articulate the new experiments that are needed.

Reviewer #1: The weaknesses detailed above needs to be addressed.

Reviewer #2: This manuscript reports a three-district implementation pilot integrating seven syndromic NTD indicators into Ghana’s Community-based Health Planning and Services (CHPS) home-visit register, with Community Health Officers (CHOs) conducting screening during routine household visits over a three-month period. The study is programmatically timely and well aligned with the WHO 2030 NTD roadmap and the UHC agenda, and it addresses a genuine operational question: whether routine community platforms can be leveraged for integrated NTD case finding and referral. The engagement of national, sub-national and district stakeholders, the use of an existing routine data tool, and the explicit attention to the referral pathway are real strengths. However, several issues require substantial attention before the manuscript can support its stated conclusions. The numerical results are internally inconsistent and the denominators underlying the reported percentages are ambiguous, which currently prevents independent verification of the descriptive findings. The proxy indicators are treated, in places, as disease-specific despite acknowledged non-specificity, risking overstatement of disease burden. The analysis is wholly descriptive with no measures of uncertainty, yet comparative and quasi-causal language is used throughout, and the policy and generalisability claims exceed what a short, purposively sampled, uncontrolled pilot can demonstrate. For these reasons I recommend a Major Revision. With transparent denominators, reconciled figures, syndromic reframing, added uncertainty estimates, and proportionate conclusions, the manuscript could make a meaningful contribution to the evidence base on integrating NTD surveillance into primary healthcare.

Major Comments

1. The study is positioned as assessing the feasibility of integrating NTD indicators into the CHPS home-visit register for national scale-up (Abstract; lines 35–38, 140–143). However, the analysis reports outputs (visits conducted, signs recorded, referrals made) without a clear denominator describing the eligible population or the expected versus achieved visit volume. The reported totals are internally inconsistent: the Abstract and Results state 1,932 home visits (lines 51, 291), yet the district figures used for referral rates sum to 1,932 only when expressed as 672 (Ga South), 409 (Nkwanta South) and 851 (Kassena Nankana West) (lines 313–318), while the facility-level visit counts implied elsewhere differ. Without a defined denominator—target households, registered population, or planned visits per CHO over the three months—the reader cannot judge whether case detection reflects genuine feasibility or simply variable field effort across districts. The authors should define the catchment denominator explicitly, report visits per CHO and per facility, and clarify whether 1,932 represents all visits or only visits in which at least one indicator was assessed. This is central to the feasibility claim and to any extrapolation toward scale-up.

2. The seven indicators are explicitly described as proxy clinical signs (lines 179–188) and the Discussion acknowledges that cases are “suspected” without confirmatory diagnostics (lines 396–398). However, the manuscript repeatedly maps single signs to specific diseases—for example, blood in urine to urogenital schistosomiasis (lines 250, 306–308) and lymphoedema to lymphatic filariasis—without addressing the well-documented non-specificity of these signs. Self-reported haematuria has limited positive predictive value outside known schistosomiasis transmission foci, and lymphoedema in this setting may reflect non-filarial causes including podoconiosis, venous disease, or trauma. Hydrocele is operationally defined as “any scrotal swelling” (line 182), which will capture numerous non-LF aetiologies. The current framing risks overstating disease-specific burden from what are, at best, syndromic screening signals. The authors should (a) reframe results as syndromic indicator frequencies rather than disease-specific prevalence, (b) explicitly discuss differential diagnoses for each indicator, and (c) quantify, where facility confirmation data exist, the proportion of referred suspected cases subsequently confirmed. This directly affects the validity of the geographical “epidemiological patterns” emphasised in the Discussion (lines 347–353).

3. Several numerical statements cannot be reconciled across the text, tables and figures, which undermines confidence in the descriptive findings. The text states skin lesions were most frequent in Ga South at 59% (386/651) followed by Nkwanta South at “approximately 25%” (lines 294–296), but Table 2 reports the Nkwanta South skin-lesion cell as 214 (23.4%) against a column total of n=651, while the district row total is n=248—so 214/248 (86%) of recorded indicators in Nkwanta South were skin lesions, a very different quantity from the 23.4% shown. The percentage bases in Table 2 are therefore ambiguous (column-wise vs row-wise vs district-population-wise), and the same ambiguity affects every cell. Similarly, the referral denominators in the text (672, 409, 851; lines 316–318) do not match the facility/visit structure described earlier, and Fig 3/Fig 4 axis values approximate but do not exactly correspond to the in-text counts. The authors must standardise all percentages to a single, clearly stated denominator, add the denominator to each table column header, and ensure exact correspondence between text, tables and figures. As presented, the central descriptive results are not independently verifiable.

4. The referral-compliance disparity is the manuscript’s headline finding (Abstract lines 57–59; Discussion lines 363–371) and is genuinely important. However, the interpretation extends beyond what a three-month, descriptive, uncontrolled pilot can support. Compliance is reported as 92.1% in Kassena Nankana West, 52% in Nkwanta South and <10% in Ga South (lines 322–325), and these differences are attributed to rurality, trust, alternative providers and transport (lines 374–381). These are plausible hypotheses, not demonstrated mechanisms, because the study collected no patient-level data on reasons for non-attendance (a limitation the authors themselves note, lines 398–401). Moreover, “compliance” appears to be ascertained from whether referred patients were recorded as attending—the verification source, time window for attendance, and handling of patients lost to follow-up are not described. The authors should specify how facility attendance was ascertained and over what period, present the compliance denominator transparently, and explicitly frame the district-level explanations as hypotheses to be tested rather than findings. Causal language linking setting to compliance should be moderated throughout.

5. The statistical approach is limited to descriptive analysis of demographic and clinical variables (lines 274–275), which is reasonable for a pilot but is not matched by appropriate caution in reporting. No confidence intervals, no denominators-with-uncertainty, and no formal comparison accompany the district contrasts, yet the text uses comparative language (“most prevalent,” “marked geographical variation,” lines 293–302) that implies inferential contrast. Given small cell counts—e.g., blood in urine n=20 total, with 15/20 in Ga South (lines 306–308), and hydrocele n=42—point estimates are highly unstable and percentages such as “75%” rest on fewer than 20 observations. The manuscript should (a) report counts alongside every percentage, (b) add 95% confidence intervals (e.g., Wilson intervals) for key proportions, (c) avoid superlative comparative claims where cell counts are small, and (d) state explicitly that observed geographical differences are descriptive and may partly reflect differential CHO effort, training intensity, or recording practice rather than true epidemiological gradients.

6. The selection of three districts across northern, middle and southern zones is presented as enhancing transferability “across diverse ecological and socio-cultural contexts” (lines 372–374), and the model is described as offering “a valuable template for other countries in sub-Saharan Africa” (lines 385–387). With one district per zone and purposive selection on the basis of NTD endemicity (lines 157–160), the design cannot support claims of representativeness within Ghana, let alone regional generalisability. District-level performance differences (e.g., 100% implementation in KNW vs lower elsewhere) are equally consistent with idiosyncratic local factors—supervision quality, individual CHO motivation, facility density—as with zone-level ecology. The authors should temper generalisability claims, frame the three districts as illustrative case settings rather than a representative sample, and explicitly acknowledge that with n=3 districts the study is underpowered to attribute outcomes to ecological zone.

7. Several conclusions are stronger than the descriptive, short-duration design warrants. The Discussion describes the integration as “a paradigm shift from population-level mass drug administration (MDA) toward individualized patient-centered care” (lines 357–359) and recommends phased national roll-out (lines 405–406). While the pilot supports the narrower claim that indicator capture is operationally feasible and acceptable to CHOs, it does not demonstrate improved case confirmation, treatment, morbidity outcomes, or cost-effectiveness—and the authors acknowledge no long-term sustainability data (lines 401–403). Recommendations such as patient-navigation systems and transport subsidies (lines 408–411) are sensible but are presented as solutions when no data on their effect were generated here. The conclusions should be reframed to distinguish what was demonstrated (feasibility of integrated syndromic data capture; high CHO uptake; a referral-completion bottleneck) from what is hypothesised (scalability, health-impact, and the efficacy of proposed remedial interventions). A cost or health-outcome claim should not be implied without supporting data.

Reviewer #3: The strength of the study has determined encouraging mechanisms for active community based case detection of NTDs

The limitation is a very low referral linkage , even in one of the study districts it was less than 10 %. One of the reasons may be sub-standard quality and access of services at PHCU, which should be part of the intervention

**********

PLOS authors have the option to publish the peer review history of their article (what does this mean?). If published, this will include your full peer review and any attached files.

If you choose “no”, your identity will remain anonymous but your review may still be made public.

**Do you want your identity to be public for this peer review?**  For information about this choice, including consent withdrawal, please see our Privacy Policy.

Reviewer #1: No

Reviewer #2: No

Reviewer #3: No

[NOTE: If reviewer comments were submitted as an attachment file, they will be attached to this email and accessible via the submission site. Please log into your account, locate the manuscript record, and check for the action link "View Attachments". If this link does not appear, there are no attachment files.]

**Figure resubmission:**

While revising your submission, we strongly recommend that you use PLOS’s NAAS tool (https://ngplosjournals.pagemajik.ai/artanalysis) to test your figure files. NAAS can convert your figure files to the TIFF file type and meet basic requirements (such as print size, resolution), or provide you with a report on issues that do not meet our requirements and that NAAS cannot fix.

After uploading your figures to PLOS’s NAAS tool - https://ngplosjournals.pagemajik.ai/artanalysis, NAAS will process the files provided and display the results in the "Uploaded Files" section of the page as the processing is complete. If the uploaded figures meet our requirements (or NAAS is able to fix the files to meet our requirements), the figure will be marked as "fixed" above. If NAAS is unable to fix the files, a red "failed" label will appear above. When NAAS has confirmed that the figure files meet our requirements, please download the file via the download option, and include these NAAS processed figure files when submitting your revised manuscript.

**Reproducibility:**

To enhance the reproducibility of your results, we recommend that authors of applicable studies deposit laboratory protocols in protocols.io, where a protocol can be assigned its own identifier (DOI) such that it can be cited independently in the future. Additionally, PLOS ONE offers an option to publish peer-reviewed clinical study protocols. Read more information on sharing protocols at https://plos.org/protocols?utm_medium=editorial-email&utm_source=authorletters&utm_campaign=protocols

Attachment Submitted filename: Comments to Authors_NTD PHC Ghana_oumer A.docx

# Author comment: Author response to Decision Letter 1

**DOI:** 10.1371/journal.pntd.0014792.r002

13 Jul 2026

Attachment Submitted filename: Response to reviewers.docx

# Peer review: Decision Letter 1

**DOI:** 10.1371/journal.pntd.0014792.r003

**Authors:** Stuart D. Blacksell, Kebede Deribe

10 Aug 2026

PNTD-D-26-00911R1

Integrating Neglected Tropical Diseases into Primary Healthcare services in Ghana: results from an initial pilot

PLOS Neglected Tropical DiseasesDear Dr. Amoako,Thank you for submitting your manuscript to PLOS Neglected Tropical Diseases. After careful consideration, we feel that it has merit but does not fully meet PLOS Neglected Tropical Diseases's publication criteria as it currently stands. Therefore, we invite you to submit a revised version of the manuscript that addresses the points raised during the review process.Please submit your revised manuscript by Sep 09 2026 11:59PM. If you will need more time than this to complete your revisions, please reply to this message or contact the journal office at plosntds@plos.org. When you're ready to submit your revision, log on to https://www.editorialmanager.com/pntd/ and select the 'Submissions Needing Revision' folder to locate your manuscript file.

Please include the following items when submitting your revised manuscript:

* A letter that responds to each point raised by the editor and reviewer(s). You should upload this letter as a separate file labeled 'Response to Reviewers'. This file does not need to include responses to any formatting updates and technical items listed in the 'Journal Requirements' section below.* A marked-up copy of your manuscript that highlights changes made to the original version. You should upload this as a separate file labeled 'Revised Manuscript with Track Changes'.* An unmarked version of your revised paper without tracked changes. You should upload this as a separate file labeled 'Manuscript'.If you would like to make changes to your financial disclosure, competing interests statement, or data availability statement, please make these updates within the submission form at the time of resubmission. Guidelines for resubmitting your figure files are available below the reviewer comments at the end of this letter.As the corresponding author, your ORCID iD is verified in the submission system and will appear in the published article. PLOS supports the use of ORCID, and we encourage all coauthors to register for an ORCID iD and use it as well. Please encourage your coauthors to verify their ORCID iD within the submission system before final acceptance, as unverified ORCID iDs will not appear in the published article. Only the individual author can complete the verification step; PLOS staff cannot verify ORCID iDs on behalf of authors.

We look forward to receiving your revised manuscript.

Kind regards,

Kebede Deribe, BSc, MPH, PhD

Guest EditorPLOS Neglected Tropical Diseases Stuart BlacksellSection EditorPLOS Neglected Tropical Diseases

Shaden Kamhawi

co-Editor-in-Chief

PLOS Neglected Tropical Diseases

orcid.org/0000-0003-4304-636XX

Paul Brindley

co-Editor-in-Chief

PLOS Neglected Tropical Diseases

orcid.org/0000-0003-1765-0002

**Reviewers' comments:** Reviewer's Responses to Questions

**Key Review Criteria Required for Acceptance?**

As you describe the new analyses required for acceptance, please consider the following:

**Methods**

-Are the objectives of the study clearly articulated with a clear testable hypothesis stated?

-Is the study design appropriate to address the stated objectives?

-Is the population clearly described and appropriate for the hypothesis being tested?

-Is the sample size sufficient to ensure adequate power to address the hypothesis being tested?

-Were correct statistical analysis used to support conclusions?

-Are there concerns about ethical or regulatory requirements being met?

Reviewer #1: The objectives of the study are clearly articulated and the study design is appropriate for the stated objectives. The sample size is also acceptable and the statistical analysis is also good enough to support the conclusions for the pilot study. No formal hypothesis testing was peformed between districts.

Reviewer #2: The revised manuscript is substantially improved and the study design is broadly appropriate for its stated purpose as a descriptive implementation pilot assessing the feasibility of integrating syndromic NTD screening into routine CHPS activities. The objectives are now more clearly framed around feasibility, case identification, referral and referral completion, rather than hypothesis testing; accordingly, the absence of a formal sample-size calculation or inferential statistical testing is acceptable for this pilot design. The population and routine household-screening approach are also better described, and the revised analysis, including clearer denominators and confidence intervals for key referral proportions, is generally appropriate to support the descriptive conclusions.

I do not consider that additional major analyses are required. The remaining issues are mainly clarifications and moderation of interpretation: the authors should clearly distinguish household visits, individuals screened, indicator observations and referred individuals; ensure that percentage-based comparisons are not described as prevalence; further temper claims regarding epidemiological patterns, generalisability and readiness for national scale-up; and clarify the denominator used for assessing implementation among trained health workers. Ethical approval and written informed consent are reported, although consent procedures for minors and handling of identifiable referral data should be briefly clarified if applicable. Subject to these minor revisions, the methods and analyses are adequate for the study objectives.

Reviewer #3: By now the objective is clearly outlined. The study design is appropriate for the study objective

justification is provided for not calculating sample size

The only thing which is missed is the justification for not including qualitative study. Probably this will be included in future studies

**********

**Results**

-Does the analysis presented match the analysis plan?

-Are the results clearly and completely presented?

-Are the figures (Tables, Images) of sufficient quality for clarity?

Reviewer #1: The results were presented were clear and complete. The figures and tables were of suffficient quality.

Reviewer #2: The authors have corrected important numerical inconsistencies, clarified the denominator used in Table 2, consolidated referral outcomes in Table 3, and added counts and 95% confidence intervals for the main referral and referral-completion proportions. The tables and remaining figures are generally adequate for communicating the findings.

A few clarifications are still warranted before acceptance. Most importantly, the Results should consistently distinguish between home visits, individuals screened, indicator observations, individuals referred, and individuals completing referral, as these are not necessarily equivalent analytical units. Table 2 percentages should also be described as the distribution of recorded syndromic indicators across districts, rather than prevalence or epidemiological rates. Finally, the denominator underlying the health-worker implementation assessment should be clarified, since 87 workers were trained whereas the district-specific implementation figures refer to 71 workers.

Reviewer #3: By now the results are clearly and completely presented

The figures and tables have sufficient quality and clarity

**********

**Conclusions**

-Are the conclusions supported by the data presented?

-Are the limitations of analysis clearly described?

-Do the authors discuss how these data can be helpful to advance our understanding of the topic under study?

-Is public health relevance addressed?

Reviewer #1: The conclusions are supported by the data presented and are acceptable. The public health relevance of integrating NTDs into the PHC has also been incorporated.

Reviewer #2: The revised Conclusions and Discussion are substantially improved and are now largely aligned with the evidence generated by this descriptive implementation pilot. The authors appropriately emphasize the operational feasibility of incorporating syndromic NTD indicators into routine CHPS home visits, strong uptake by trained health workers, and the important gap between referral and completed facility attendance. The limitations section has also been strengthened to acknowledge the short implementation period, absence of population-level denominators, reliance on non-specific syndromic indicators without diagnostic confirmation, small numbers for some indicators, and the inability to investigate determinants of referral completion or longer-term sustainability.

The manuscript also discusses the contribution of these findings to understanding how an existing community health platform may support integrated NTD case detection and linkage to care, and the public health relevance is clearly articulated in relation to PHC, UHC and the NTD control and elimination agenda. Some conclusions should nevertheless be further moderated: statements suggesting readiness for wider or national implementation, strong transferability across Ghana, or broader regional generalisability remain stronger than can be supported by three purposively selected districts and a three-month pilot. These should be reframed as justification for broader prospective evaluation rather than demonstrated scalability.

Reviewer #3: By now the comments have been addressed. The interpretation language has been revised throughout to ensure consistency with a descriptive analytical approach (as a pilot study)) and to avoid overinterpretation of the findings. However, I still believe there should be engagement of specialists like dermatologists to confirm the diagnosis made by CHOs

**********

**Editorial and Data Presentation Modifications?**

Use this section for editorial suggestions as well as relatively minor modifications of existing data that would enhance clarity. If the only modifications needed are minor and/or editorial, you may wish to recommend “Minor Revision” or “Accept”.

Reviewer #1: None

Reviewer #2: I recommend Minor Revision. The manuscript has improved substantially and the remaining issues are primarily editorial or involve clarification of existing data rather than additional analyses.

The authors should ensure consistent terminology throughout, particularly distinguishing household visits, individuals screened, syndromic indicator observations, individuals referred, and referral completions. This distinction is important because one household visit may include several individuals and one individual may contribute more than one indicator. Relatedly, percentages in Table 2 should be described consistently as the distribution of recorded syndromic indicators across districts, rather than as prevalence or disease-specific rates.

The denominator used for the health-worker implementation assessment should also be clarified. Although 87 workers were trained overall, the district-specific implementation figures sum to 71 assessed workers. The manuscript should explain who was included in this assessment and why the denominator differs from the total trained population.

A final editorial revision should moderate residual language implying epidemiological patterns, national scalability, or broad generalisability, and ensure that suggested interventions to improve referral completion are presented as potential strategies requiring evaluation rather than demonstrated solutions. The remaining figure/table presentation is generally adequate, although Table 2 and Table 3 captions and footnotes should make their denominators and analytical units explicit.

Finally, a careful language and reference-formatting check is warranted to correct remaining grammatical inconsistencies and incomplete reference entries. Subject to these relatively minor revisions, the manuscript would be suitable for acceptance.

Reviewer #3: In view of the authors' thorough and diligent responses to the reviewers' comments and their explicit acknowledgment of the study's limitations with respect to the generalizability of the findings, I have decided to accept this manuscript for publication

**********

**Summary and General Comments**

Use this section to provide overall comments, discuss strengths/weaknesses of the study, novelty, significance, general execution and scholarship. You may also include additional comments for the author, including concerns about dual publication, research ethics, or publication ethics. If requesting major revision, please articulate the new experiments that are needed.

Reviewer #1: Acceptable for publication.

Reviewer #2: The manuscript addresses an important implementation question on integrating syndromic NTD screening and referral into Ghana’s CHPS platform. Its main strengths are the pragmatic use of an existing primary healthcare system, stakeholder engagement, strong CHO uptake, and the identification of a substantial referral-completion gap. The revised manuscript is considerably improved, particularly in the description of screening procedures, referral ascertainment, and study limitations.

The remaining issues are mainly minor. The authors should clearly distinguish household visits, individuals screened, indicator observations, referrals and completed referrals; avoid describing syndromic indicator distributions as prevalence or epidemiological patterns; clarify the denominator used for the health-worker implementation assessment; and further moderate claims regarding scalability and generalisability.

Overall, the study provides useful operational evidence with clear public health relevance. I recommend Minor Revision.

Reviewer #3: We would like to extend our sincere gratitude to the authors for their thorough dedication in addressing nearly all of the reviewers’ comments. We believe this pilot study will serve as a critical foundation for a future mixed-methods implementation study, ultimately generating the necessary evidence to support the integration of NTD care into primary healthcare (PHC) systems.

**********

PLOS authors have the option to publish the peer review history of their article (what does this mean?). If published, this will include your full peer review and any attached files.

If you choose “no”, your identity will remain anonymous but your review may still be made public.

**Do you want your identity to be public for this peer review?**  For information about this choice, including consent withdrawal, please see our Privacy Policy.

Reviewer #1: No

Reviewer #2: No

Reviewer #3: No

[NOTE: If reviewer comments were submitted as an attachment file, they will be attached to this email and accessible via the submission site. Please log into your account, locate the manuscript record, and check for the action link "View Attachments". If this link does not appear, there are no attachment files.] **Figure resubmission:**   While revising your submission, we strongly recommend that you use PLOS’s NAAS tool (https://ngplosjournals.pagemajik.ai/artanalysis) to test your figure files. NAAS can convert your figure files to the TIFF file type and meet basic requirements (such as print size, resolution), or provide you with a report on issues that do not meet our requirements and that NAAS cannot fix.

After uploading your figures to PLOS’s NAAS tool - https://ngplosjournals.pagemajik.ai/artanalysis, NAAS will process the files provided and display the results in the "Uploaded Files" section of the page as the processing is complete. If the uploaded figures meet our requirements (or NAAS is able to fix the files to meet our requirements), the figure will be marked as "fixed" above. If NAAS is unable to fix the files, a red "failed" label will appear above. When NAAS has confirmed that the figure files meet our requirements, please download the file via the download option, and include these NAAS processed figure files when submitting your revised manuscript. **Reproducibility:**  To enhance the reproducibility of your results, we recommend that authors of applicable studies deposit laboratory protocols in protocols.io, where a protocol can be assigned its own identifier (DOI) such that it can be cited independently in the future. Additionally, PLOS ONE offers an option to publish peer-reviewed clinical study protocols. Read more information on sharing protocols at https://plos.org/protocols?utm_medium=editorial-email&utm_source=authorletters&utm_campaign=protocols

# Author comment: Author response to Decision Letter 2

**DOI:** 10.1371/journal.pntd.0014792.r004

11 Aug 2026

Attachment Submitted filename: Response to review comments.docx

# Peer review: Decision Letter 2

**DOI:** 10.1371/journal.pntd.0014792.r005

**Authors:** Kebede Deribe, Elsio A Wunder Jr

1 Sep 2026

PNTD-D-26-00911R2Integrating Neglected Tropical Diseases into Primary Healthcare services in Ghana: results from an initial pilotPLOS Neglected Tropical DiseasesDear Dr. Amoako,Thank you for submitting your manuscript to PLOS Neglected Tropical Diseases. After careful consideration, we feel that it has merit but does not fully meet PLOS Neglected Tropical Diseases's publication criteria as it currently stands. Therefore, we invite you to submit a revised version of the manuscript that addresses the points raised during the review process.Please submit your revised manuscript by Oct 01 2026 11:59PM. If you will need more time than this to complete your revisions, please reply to this message or contact the journal office at plosntds@plos.org. When you're ready to submit your revision, log on to https://www.editorialmanager.com/pntd/ and select the 'Submissions Needing Revision' folder to locate your manuscript file.

Please include the following items when submitting your revised manuscript:* A letter that responds to each point raised by the editor and reviewer(s). You should upload this letter as a separate file labeled 'Response to Reviewers'. This file does not need to include responses to any formatting updates and technical items listed in the 'Journal Requirements' section below.* A marked-up copy of your manuscript that highlights changes made to the original version. You should upload this as a separate file labeled 'Revised Manuscript with Track Changes'.* An unmarked version of your revised paper without tracked changes. You should upload this as a separate file labeled 'Manuscript'.If you would like to make changes to your financial disclosure, competing interests statement, or data availability statement, please make these updates within the submission form at the time of resubmission. Guidelines for resubmitting your figure files are available below the reviewer comments at the end of this letter.As the corresponding author, your ORCID iD is verified in the submission system and will appear in the published article. PLOS supports the use of ORCID, and we encourage all coauthors to register for an ORCID iD and use it as well. Please encourage your coauthors to verify their ORCID iD within the submission system before final acceptance, as unverified ORCID iDs will not appear in the published article. Only the individual author can complete the verification step; PLOS staff cannot verify ORCID iDs on behalf of authors.We look forward to receiving your revised manuscript.Kind regards,Kebede Deribe, BSc, MPH, PhDGuest EditorPLOS Neglected Tropical DiseasesStuart BlacksellSection EditorPLOS Neglected Tropical Diseases

Shaden Kamhawi

co-Editor-in-Chief

PLOS Neglected Tropical Diseases

orcid.org/0000-0003-4304-636XX

Paul Brindley

co-Editor-in-Chief

PLOS Neglected Tropical Diseases

orcid.org/0000-0003-1765-0002

**Reviewers' comments:** Reviewer's Responses to Questions

**Key Review Criteria Required for Acceptance?**

As you describe the new analyses required for acceptance, please consider the following:

**Methods**

-Are the objectives of the study clearly articulated with a clear testable hypothesis stated?

-Is the study design appropriate to address the stated objectives?

-Is the population clearly described and appropriate for the hypothesis being tested?

-Is the sample size sufficient to ensure adequate power to address the hypothesis being tested?

-Were correct statistical analysis used to support conclusions?

-Are there concerns about ethical or regulatory requirements being met?

Reviewer #2: The Methods are generally appropriate for the study’s stated purpose as a descriptive implementation pilot, and the objectives are sufficiently clear within that feasibility framework. A formal testable hypothesis, sample-size calculation, or inferential comparison is not essential for this type of pilot, provided the conclusions remain descriptive and proportionate to the design. The population and routine CHPS screening procedures are adequately described, and the statistical approach is broadly appropriate.

The main issue still requiring clarification is the unit of analysis and denominator structure. The manuscript should clearly distinguish household visits, individuals screened, indicator observations, individuals referred and referral completions, and ensure that referral proportions are calculated using compatible numerator and denominator units. The denominator for the health-worker implementation assessment should also be reconciled. No major ethical concerns are apparent, although consent procedures for minors and handling of identifiable referral data should be clarified if applicable.

Overall, no major new analyses are required; the remaining issues are methodological clarifications that should be resolved before acceptance.

**********

**Results**

-Does the analysis presented match the analysis plan?

-Are the results clearly and completely presented?

-Are the figures (Tables, Images) of sufficient quality for clarity?

Reviewer #2: The Results are broadly consistent with the stated descriptive analysis plan, and the revised tables are clearer than in the original submission. However, some numerical presentation issues remain and should be resolved before acceptance. In particular, the manuscript still mixes household visits, indicator observations, referred individuals and referral completions as analytical units, which makes some derived proportions difficult to interpret.

The main figures requiring clarification are:

- 1,932 home visits

- 1,117 indicator observations

- 615 referrals

- 415 completed referrals overall, derived from 4 + 130 + 281

- District referral proportions reported as 62/672 = 9.2%, 248/409 = 60.6%, and 305/851 = 35.8%

The key concern is that these referral proportions use home visits as the denominator, while the numerator is described elsewhere as individuals referred. Since several household members could be screened during one visit and one individual could contribute more than one indicator, numerator and denominator may represent different units. This needs to be clarified or recalculated using a compatible denominator.

A second issue is Table 2. Its footnote correctly states that percentages represent the proportion of all recorded observations for each indicator contributed by each district and are not prevalence estimates. However, the text still describes figures such as 59.3% skin lesions in Ga South, 81.6% visible deformities in Ga South, and 75% blood in urine in Ga South using terms such as “prevalence” or “most prevalent.” These are distributional proportions, not district prevalence estimates, and the wording should be corrected accordingly.

Finally, the health-worker implementation results remain internally unclear: 87 workers were trained, whereas the district-specific implementation denominators are 21 + 28 + 22 = 71, with 63 reported as implementing. The manuscript should explain why 16 trained workers are not represented in this assessment and define exactly which cadres are included.

Overall, the tables and figures are of acceptable quality, but these inconsistencies in analytical units and denominators should be addressed before publication.

**********

**Conclusions**

-Are the conclusions supported by the data presented?

-Are the limitations of analysis clearly described?

-Do the authors discuss how these data can be helpful to advance our understanding of the topic under study?

-Is public health relevance addressed?

Reviewer #2: The Conclusions are generally supported by the data insofar as they emphasize the operational feasibility of incorporating syndromic NTD indicators into CHPS activities, strong uptake by CHOs, and the substantial referral-completion gap. The limitations are now clearly described, including the lack of population-level denominators, absence of diagnostic confirmation, possible misclassification, small numbers for some indicators, and the short duration of the pilot. The manuscript also explains the public health relevance of strengthening community-based NTD detection and linkage to care within PHC and UHC.

However, some statements still exceed the evidence. Claims concerning “epidemiological patterns,” readiness for wider or national implementation, enhanced transferability across Ghana, and broader generalisability to sub-Saharan Africa should be further moderated. Given the purposive selection of only three districts, absence of representative population denominators, and lack of diagnostic validation, the findings are better framed as proof-of-concept operational evidence that justifies broader evaluation, rather than evidence of established scalability or generalisability.

Overall, the conclusions are close to acceptable, but these overinterpretations should be corrected before publication.

**********

**Editorial and Data Presentation Modifications?**

Use this section for editorial suggestions as well as relatively minor modifications of existing data that would enhance clarity. If the only modifications needed are minor and/or editorial, you may wish to recommend “Minor Revision” or “Accept”.

Reviewer #2: I recommend Minor Revision. The manuscript is substantially improved, and the remaining issues are primarily editorial and related to clarification of existing data rather than requiring new fieldwork or major analyses.

The authors should ensure consistent use of analytical units throughout the Results, clearly distinguishing home visits, individuals screened, indicator observations, individuals referred, and referral completions. In particular, the referral proportions in Table 3 should be checked to ensure that numerator and denominator refer to compatible units. The health-worker implementation denominator should also be clarified, since 87 workers were trained but the district-specific implementation assessment includes only 71.

Table 2 is now much clearer, but the accompanying text should avoid terms such as “prevalence” or “epidemiological patterns”, because the reported percentages represent the distribution of syndromic indicator observations across districts rather than population-based prevalence estimates. Similarly, statements regarding national scale-up, transferability across Ghana, or generalisability to other countries should be further moderated and framed as areas for future evaluation.

A final editorial review is recommended to address several residual language and formatting issues. Examples include subject–verb agreement (“versions… was piloted”; “disparities… underscores”), grammatical errors (“could possibility result”), inconsistent terminology (“primary healthcare/primary health care”; “sub-national/subnational”), and continued use of “prevalence” for percentages that Table 2 correctly defines as distributions of syndromic observations. Reference 23 is also incomplete (“2021 Census reports:[”), and the formatting of organizational/online references should be standardized. These are minor editorial issues but should be corrected before publication.

**********

**Summary and General Comments**

Use this section to provide overall comments, discuss strengths/weaknesses of the study, novelty, significance, general execution and scholarship. You may also include additional comments for the author, including concerns about dual publication, research ethics, or publication ethics. If requesting major revision, please articulate the new experiments that are needed.

Reviewer #2: The manuscript addresses a relevant implementation question and provides useful operational evidence on incorporating syndromic NTD screening into Ghana’s CHPS platform. Its main strengths are the pragmatic use of an existing primary healthcare structure, stakeholder engagement, CHO participation, and the identification of an important referral-completion gap. The revised manuscript is substantially improved compared with the original submission, particularly in the description of screening procedures, referral ascertainment, presentation of referral outcomes, and acknowledgement of key limitations.

The remaining weaknesses are mainly related to clarity and interpretation rather than study execution. The authors should resolve the unit-of-analysis issue across home visits, individuals screened, indicator observations and referrals; clarify the denominator for the health-worker implementation assessment; avoid describing syndromic indicator distributions as prevalence or epidemiological patterns; and further temper claims regarding scalability and generalisability. No new experiments or major analyses are required.

Overall, the study is programmatically relevant and potentially publishable after these final clarifications. I recommend Minor Revision.

**********

PLOS authors have the option to publish the peer review history of their article (what does this mean?). If published, this will include your full peer review and any attached files.

If you choose “no”, your identity will remain anonymous but your review may still be made public.

**Do you want your identity to be public for this peer review?**  For information about this choice, including consent withdrawal, please see our Privacy Policy.

Reviewer #2: No

[NOTE: If reviewer comments were submitted as an attachment file, they will be attached to this email and accessible via the submission site. Please log into your account, locate the manuscript record, and check for the action link "View Attachments". If this link does not appear, there are no attachment files.]**Figure resubmission:** While revising your submission, we strongly recommend that you use PLOS’s NAAS tool (https://ngplosjournals.pagemajik.ai/artanalysis) to test your figure files. NAAS can convert your figure files to the TIFF file type and meet basic requirements (such as print size, resolution), or provide you with a report on issues that do not meet our requirements and that NAAS cannot fix.

After uploading your figures to PLOS’s NAAS tool - https://ngplosjournals.pagemajik.ai/artanalysis, NAAS will process the files provided and display the results in the "Uploaded Files" section of the page as the processing is complete. If the uploaded figures meet our requirements (or NAAS is able to fix the files to meet our requirements), the figure will be marked as "fixed" above. If NAAS is unable to fix the files, a red "failed" label will appear above. When NAAS has confirmed that the figure files meet our requirements, please download the file via the download option, and include these NAAS processed figure files when submitting your revised manuscript.**Reproducibility:** To enhance the reproducibility of your results, we recommend that authors of applicable studies deposit laboratory protocols in protocols.io, where a protocol can be assigned its own identifier (DOI) such that it can be cited independently in the future. Additionally, PLOS ONE offers an option to publish peer-reviewed clinical study protocols. Read more information on sharing protocols at https://plos.org/protocols?utm_medium=editorial-email&utm_source=authorletters&utm_campaign=protocols

# Author comment: Author response to Decision Letter 3

**DOI:** 10.1371/journal.pntd.0014792.r006

5 Sep 2026

Attachment Submitted filename: Response_to_review_comments_auresp_3.docx

# Peer review: Decision Letter 3

**DOI:** 10.1371/journal.pntd.0014792.r007

**Authors:** Kebede Deribe, Elsio A Wunder Jr

28 Sep 2026

PNTD-D-26-00911R3

Integrating Neglected Tropical Diseases into Primary Healthcare services in Ghana: results from an initial pilot

PLOS Neglected Tropical DiseasesDear Dr. Amoako,Thank you for submitting your manuscript to PLOS Neglected Tropical Diseases. After careful consideration, we feel that it has merit but does not fully meet PLOS Neglected Tropical Diseases's publication criteria as it currently stands. Therefore, we invite you to submit a revised version of the manuscript that addresses the points raised during the review process.Please submit your revised manuscript by Oct 28 2026 11:59PM. If you will need more time than this to complete your revisions, please reply to this message or contact the journal office at plosntds@plos.org. When you're ready to submit your revision, log on to https://www.editorialmanager.com/pntd/ and select the 'Submissions Needing Revision' folder to locate your manuscript file.Please include the following items when submitting your revised manuscript:* A letter that responds to each point raised by the editor and reviewer(s). You should upload this letter as a separate file labeled 'Response to Reviewers'. This file does not need to include responses to any formatting updates and technical items listed in the 'Journal Requirements' section below.* A marked-up copy of your manuscript that highlights changes made to the original version. You should upload this as a separate file labeled 'Revised Manuscript with Track Changes'.* An unmarked version of your revised paper without tracked changes. You should upload this as a separate file labeled 'Manuscript'.If you would like to make changes to your financial disclosure, competing interests statement, or data availability statement, please make these updates within the submission form at the time of resubmission. Guidelines for resubmitting your figure files are available below the reviewer comments at the end of this letter.As the corresponding author, your ORCID iD is verified in the submission system and will appear in the published article. PLOS supports the use of ORCID, and we encourage all coauthors to register for an ORCID iD and use it as well. Please encourage your coauthors to verify their ORCID iD within the submission system before final acceptance, as unverified ORCID iDs will not appear in the published article. Only the individual author can complete the verification step; PLOS staff cannot verify ORCID iDs on behalf of authors.

We look forward to receiving your revised manuscript.Kind regards,Kebede Deribe, BSc, MPH, PhDGuest EditorPLOS Neglected Tropical DiseasesStuart BlacksellSection EditorPLOS Neglected Tropical Diseases

Shaden Kamhawi

co-Editor-in-Chief

PLOS Neglected Tropical Diseases

orcid.org/0000-0003-4304-636XX

Paul Brindley

co-Editor-in-Chief

PLOS Neglected Tropical Diseases

orcid.org/0000-0003-1765-0002

**Reviewers' comments:** Reviewer's Responses to Questions

**Key Review Criteria Required for Acceptance?**

As you describe the new analyses required for acceptance, please consider the following:

**Methods**

-Are the objectives of the study clearly articulated with a clear testable hypothesis stated?

-Is the study design appropriate to address the stated objectives?

-Is the population clearly described and appropriate for the hypothesis being tested?

-Is the sample size sufficient to ensure adequate power to address the hypothesis being tested?

-Were correct statistical analysis used to support conclusions?

-Are there concerns about ethical or regulatory requirements being met?

Reviewer #2: The Methods are now appropriate for the study’s stated purpose as a descriptive implementation pilot. The objectives, study population, routine CHPS screening procedures, descriptive analytical approach, and ethical safeguards are sufficiently described, and no additional major analyses or sample-size calculations are required for acceptance. The lack of formal hypothesis testing is acceptable given the feasibility-focused design.

The previous concerns regarding consent for minors and handling of identifiable referral data have also been addressed. The only remaining methodological issue is largely terminological: the referral measure should be described consistently as referrals generated relative to household visits, rather than implying an individual-level referral proportion. This is now explained in the Results and does not require further analysis.

**********

**Results**

-Does the analysis presented match the analysis plan?

-Are the results clearly and completely presented?

-Are the figures (Tables, Images) of sufficient quality for clarity?

Reviewer #2: The Results are now consistent with the descriptive analysis plan and are presented clearly overall. The revised text distinguishes indicator observations from prevalence estimates, explains the referral metric more transparently, and presents referral completion with appropriate counts and confidence intervals. The tables and figures are generally of sufficient quality to support interpretation.

Only minor wording could still be improved, particularly by describing the referral measure consistently as referrals generated relative to household visits rather than as an individual-level referral proportion. This does not require additional analysis and would not prevent acceptance. See comments provided to authors.

**********

**Conclusions**

-Are the conclusions supported by the data presented?

-Are the limitations of analysis clearly described?

-Do the authors discuss how these data can be helpful to advance our understanding of the topic under study?

-Is public health relevance addressed?

Reviewer #2: The Conclusions are now well aligned with the data presented. The authors appropriately emphasize the feasibility of incorporating syndromic NTD indicators into routine CHPS activities, the strong engagement of CHOs, and the importance of addressing referral-completion gaps. The limitations are clearly acknowledged, including the short duration, absence of population-level denominators, lack of diagnostic confirmation, possible misclassification, and limited ability to assess determinants of referral completion or long-term sustainability.

The manuscript also explains how these findings contribute to the operational evidence base for integrating NTD detection and referral into primary health care, and the public health relevance is clearly articulated in relation to UHC and NTD control and elimination. The revised Conclusion is appropriately cautious and frames the findings as proof-of-concept evidence requiring evaluation in larger studies. I do not consider further substantive revision necessary.

**********

**Editorial and Data Presentation Modifications?**

Use this section for editorial suggestions as well as relatively minor modifications of existing data that would enhance clarity. If the only modifications needed are minor and/or editorial, you may wish to recommend “Minor Revision” or “Accept”.

Reviewer #2: I recommend Accept with minor editorial corrections. The manuscript has improved substantially across successive revisions, and the remaining issues do not affect the validity of the findings or require additional analyses.

A few minor editorial refinements would further improve clarity: consistently describe the referral metric as referrals generated relative to household visits; clarify that the implementation percentages presented in Figure 3 refer to CHOs; remove or soften the remaining “mixed-methods” wording unless qualitative methods/results are formally presented; and ensure that residual statements about transferability, generalisability, and national scale-up remain aligned with the cautious framing adopted in the revised Conclusion. A final proofread for grammar and terminology would also be appropriate. Overall, these are editorial points only, and I consider the manuscript suitable for publication.

**********

**Summary and General Comments**

Use this section to provide overall comments, discuss strengths/weaknesses of the study, novelty, significance, general execution and scholarship. You may also include additional comments for the author, including concerns about dual publication, research ethics, or publication ethics. If requesting major revision, please articulate the new experiments that are needed.

Reviewer #2: I identified only a few residual inconsistencies, which I would treat as editorial:

- Referral terminology: because several people could be screened during one household visit, “referrals per household visit” or “referrals per 100 household visits” would be technically clearer than “proportion of home visits leading to referrals” unless the authors can confirm a maximum of one referral per visit.

- Implementation denominator: the revised paragraph says the assessment included CHOs, public health nurses and disease control officers, but the district denominators reported (21, 25 and 18) correspond to the 64 CHOs, and Figure 3 is explicitly labelled as CHOs. The text should simply state that the implementation percentages presented refer to CHOs.

- Some earlier wording remains stronger than the revised Conclusion. The Discussion still says the three districts “enhance the transferability” of the findings, that task-sharing is “highly generalizable,” and refers to national scale-up/phased national roll-out. Given that the Conclusion now appropriately calls for larger studies to evaluate scalability, these residual phrases could be softened for consistency.

- The Discussion still calls the monitoring a “mixed-methods monitoring approach”, although the limitations state that the study did not include a qualitative component. I would simply remove “mixed-methods” or describe this as monitoring incorporating qualitative feedback.

**********

PLOS authors have the option to publish the peer review history of their article (what does this mean?). If published, this will include your full peer review and any attached files.

If you choose “no”, your identity will remain anonymous but your review may still be made public.

**Do you want your identity to be public for this peer review?**  For information about this choice, including consent withdrawal, please see our Privacy Policy.

Reviewer #2: No

[NOTE: If reviewer comments were submitted as an attachment file, they will be attached to this email and accessible via the submission site. Please log into your account, locate the manuscript record, and check for the action link "View Attachments". If this link does not appear, there are no attachment files.]

**Figure resubmission:**  While revising your submission, we strongly recommend that you use PLOS’s NAAS tool (https://ngplosjournals.pagemajik.ai/artanalysis) to test your figure files. NAAS can convert your figure files to the TIFF file type and meet basic requirements (such as print size, resolution), or provide you with a report on issues that do not meet our requirements and that NAAS cannot fix.

After uploading your figures to PLOS’s NAAS tool - https://ngplosjournals.pagemajik.ai/artanalysis, NAAS will process the files provided and display the results in the "Uploaded Files" section of the page as the processing is complete. If the uploaded figures meet our requirements (or NAAS is able to fix the files to meet our requirements), the figure will be marked as "fixed" above. If NAAS is unable to fix the files, a red "failed" label will appear above. When NAAS has confirmed that the figure files meet our requirements, please download the file via the download option, and include these NAAS processed figure files when submitting your revised manuscript.**Reproducibility:** To enhance the reproducibility of your results, we recommend that authors of applicable studies deposit laboratory protocols in protocols.io, where a protocol can be assigned its own identifier (DOI) such that it can be cited independently in the future. Additionally, PLOS ONE offers an option to publish peer-reviewed clinical study protocols. Read more information on sharing protocols at https://plos.org/protocols?utm_medium=editorial-email&utm_source=authorletters&utm_campaign=protocol

# Author comment: Author response to Decision Letter 4

**DOI:** 10.1371/journal.pntd.0014792.r008

28 Sep 2026

Attachment Submitted filename: Response_to_reviewers_auresp_4.docx

# Editor report: Decision Letter 4

**DOI:** 10.1371/journal.pntd.0014792.r009

**Authors:** Kebede Deribe, Elsio A Wunder Jr

29 Sep 2026

Dear Amoako,

We are pleased to inform you that your manuscript 'Integrating Neglected Tropical Diseases into Primary Healthcare services in Ghana: results from an initial pilot' has been provisionally accepted for publication in PLOS Neglected Tropical Diseases.

Before your manuscript can be formally accepted you will need to complete some formatting changes, which you will receive in a follow up email. A member of our team will be in touch with a set of requests.

Please note that your manuscript will not be scheduled for publication until you have made the required changes, so a swift response is appreciated.

IMPORTANT: The editorial review process is now complete. PLOS will only permit corrections to spelling, formatting or significant scientific errors from this point onwards. Requests for major changes, or any which affect the scientific understanding of your work, will cause delays to the publication date of your manuscript.

Should you, your institution's press office or the journal office choose to press release your paper, you will automatically be opted out of early publication. We ask that you notify us now if you or your institution is planning to press release the article. All press must be co-ordinated with PLOS.

Thank you again for supporting Open Access publishing; we are looking forward to publishing your work in PLOS Neglected Tropical Diseases.

Best regards,

Kebede Deribe, BSc, MPH, PhD

Guest Editor

PLOS Neglected Tropical Diseases

Stuart Blacksell

Section Editor

PLOS Neglected Tropical Diseases

Shaden Kamhawi

co-Editor-in-Chief

PLOS Neglected Tropical Diseases

orcid.org/0000-0003-4304-636XX

Paul Brindley

co-Editor-in-Chief

PLOS Neglected Tropical Diseases

orcid.org/0000-0003-1765-0002

***********************************************************

# Editor report: Acceptance letter

**DOI:** 10.1371/journal.pntd.0014792.r010

**Authors:** Kebede Deribe, Elsio A Wunder Jr

Dear Amoako,

We are delighted to inform you that your manuscript, "Integrating Neglected Tropical Diseases into Primary Healthcare services in Ghana: results from an initial pilot," has been formally accepted for publication in PLOS Neglected Tropical Diseases.

We have now passed your article onto the PLOS Production Department who will complete the rest of the publication process. All authors will receive a confirmation email upon publication.

The corresponding author will soon be receiving a typeset proof for review, to ensure errors have not been introduced during production. Please review the PDF proof of your manuscript carefully, as this is the last chance to correct any scientific or type-setting errors. Please note that major changes, or those which affect the scientific understanding of the work, will likely cause delays to the publication date of your manuscript. Note: Proofs for Front Matter articles (Editorial, Viewpoint, Symposium, Review, etc...) are generated on a different schedule and may not be made available as quickly.

Soon after your final files are uploaded, the early version of your manuscript will be published online unless you opted out of this process. The date of the early version will be your article's publication date. The final article will be published to the same URL, and all versions of the paper will be accessible to readers.

For Research Articles, you will receive an invoice from PLOS for your publication fee after your manuscript has reached the completed accept phase. If you receive an email requesting payment before acceptance or for any other service, this may be a phishing scheme. Learn how to identify phishing emails and protect your accounts at https://explore.plos.org/phishing.

Thank you again for supporting open-access publishing; we are looking forward to publishing your work in PLOS Neglected Tropical Diseases.

Best regards,

Shaden Kamhawi

co-Editor-in-Chief

PLOS Neglected Tropical Diseases

Paul Brindley

co-Editor-in-Chief

PLOS Neglected Tropical Diseases
